# Correlation minor norms, entanglement detection and discord

**DOI:** 10.1038/s41598-021-82303-3

**Published:** 2021-02-02

**Authors:** Bar Y. Peled, Amit Te’eni, Avishy Carmi, Eliahu Cohen

**Affiliations:** 1 Center for Quantum Information Science and Technology and Faculty of Engineering Sciences, Ben-Gurion University of the Negev, Beersheba, 8410501 Israel; 2 Faculty of Engineering and the Institute of Nanotechnology and Advanced Materials, Bar Ilan University, Ramat Gan, 5290002 Israel

**Keywords:** Quantum physics, Quantum information, Quantum mechanics

## Abstract

In this paper we develop an approach for detecting entanglement, which is based on measuring quantum correlations and constructing a correlation matrix. The correlation matrix is then used for defining a family of parameters, named Correlation Minor Norms, which allow one to detect entanglement. This approach generalizes the computable cross-norm or realignment (CCNR) criterion, and moreover requires measuring a state-independent set of operators. Furthermore, we illustrate a scheme which yields for each Correlation Minor Norm a separable state that maximizes it. The proposed entanglement detection scheme is believed to be advantageous in comparison to other methods because correlations have a simple, intuitive meaning and in addition they can be directly measured in experiment. Moreover, it is demonstrated to be stronger than the CCNR criterion. We also illustrate the relation between the Correlation Minor Norm and entanglement entropy for pure states. Finally, we discuss the relation between the Correlation Minor Norm and quantum discord. We demonstrate that the CMN may be used to define a new measure for quantum discord.

## Introduction

The last three decades have seen significant advancement in development of promising quantum technologies, both from theoretical and practical aspects. These technologies often utilize quantum entanglement in order to gain advantage compared to classical technologies. Thus, the practical ability to detect entanglement is essential for the advancement of quantum technologies. Entanglement detection in many-body quantum systems is also of major interest^1–4^, as well as quantum correlations in various physical settings such as those occurring in quantum optics^5–8^, solid-state physics^9–11^ and atomic physics^12–17^.

This has led researchers to seek simple ways to detect entanglement, preferably, ones which may be used in practice. For example, the Peres–Horodecki criterion^18^ is a necessary condition for a state to be separable; however, it is sufficient only in the $2 \times 2$ and $2 \times 3$ dimensional cases^19,20^.

Another important concept is an entanglement witness, which is a measurable quantum property (i.e. a bounded Hermitian operator), such that its expectation value is always non-negative for separable states^19^. For any entangled state, there is at least one entanglement witness which would achieve a negative expectation value in this state. Alas, to use an entanglement witness in order to detect entanglement, one must measure a specific operator tailored to the state. An approach to quantify entanglement using entanglement witnesses can be found in^21^.

In^22–27^, a construction of a quantum correlation matrix was demonstrated, and it was shown that this matrix may be utilized to detect entanglement. In^28,29^, a quantum correlation matrix has allowed the authors to derive generalized uncertainty relations, as well as a novel approach for finding bounds on nonlocal correlations. This matrix is the correlation matrix of a vector of quantum observables; thus, it may have complex entries. In^30^ it was demonstrated that such a matrix allows one to construct new Bell parameters and find their Tsirelson bounds. Another approach for Bell parameters based on covariance can be found in^31^.

Indeed, quantum correlations are subtly related to entanglement, e.g. pure product states are always uncorrelated. This is not true for mixed states: separable mixed states may admit quantum correlations between remote parties^32^. These correlations are due to noncommutativity of quantum operators; hence, they allude to a different quantum property aside of entanglement, known as quantum discord^32–37^. Since quantum discord is generally hard to compute when using its original definition, researchers have examined other discord measures which are more computationally tractable—most notably, geometric quantum discord^38,39^.

In^40,41^, an approach for detecting entanglement using symmetric polynomials of the state’s Schimdt coefficients has been studied. It was shown to be a generalization of the well-known CCNR criterion (computable cross-norm or realignment; first defined in^42,43^), according to which the sum of all Schmidt coefficients is no greater than 1 for any separable state. The symmetric polynomial approach equips each one of these polynomials with some upper bound, and if the polynomial exceeds its bound then it follows that the state is entangled. Therefore, the sum of all Schmidt coefficients with the upper bound 1 is a special case of this approach.

In this paper, we construct for a given quantum state its quantum correlation matrix, and examine the norms of its *compound* matrices. Since the compound matrix in our case is constructed from minors of a certain correlation matrix, we call the proposed entanglement detectors “Correlation Minor Norms”. Seeing that these norms are invariant under orthogonal transformations of the observables, they can be regarded as a family of physical scalars which can be readily derived from bipartite correlations. Next, for each Correlation Minor Norm (CMN) we find an upper bound, such that if the CMN exceeds this bound it is implied that the state is entangled. Our proposed method is shown to generalize the symmetric polynomial approach. We also provide results and conjectures regarding the states that saturate the bounds. Moreover, we explore how the CMN relates to entanglement entropy. Next, we construct a novel measure for quantum discord based on the CMN. In a particular case, it is identical to geometric quantum discord. We conclude by discussing possible generalizations for multipartite scenarios.

## Construction of the correlation matrix

Let two remote parties, Alice and Bob, share a quantum system in ${\mathscr {H}}_A \otimes {\mathscr {H}}_B$, the tensor product of Hilbert spaces. Denote $d_A :=\dim {\mathscr {H}}_A , d_B :=\dim {\mathscr {H}}_B$, and let ${\varvec{A}} :=\left\{ A_i \right\} _{i=1}^{d_A^2}$ be an orthonormal basis of the (real) vector space of $d_A \times d_A$ Hermitian operators, w.r.t. the Hilbert-Schmidt inner product. Similarly, ${\varvec{B}} :=\left\{ B_j \right\} _{j=1}^{d_B^2}$ is an orthonormal basis of the $d_B \times d_B$ Hermitian matrices. Note that such a basis always exists, since the real vector space of $n \times n$ Hermitian matrices is simply the real Lie algebra ${\mathfrak {u}} \left( n \right)$, which is known to have dimension $n^2$. Here we regard ${\mathfrak {u}} \left( d_{A} \right) , {\mathfrak {u}} \left( d_{B} \right)$ simply as inner product spaces, ignoring their Lie algebraic properties. Consequentially, we require the normalization $\mathrm {tr}\left( A_i A_j\right) = \delta _{ij}$ (and similarly for Bob)—without the factor of 2, which is normally taken to make the structure constants more convenient. For example, for $d = 3$ one could take $A_9 = \frac{1}{\sqrt{3}} \mathbb {1}$ and $A_i = \frac{1}{\sqrt{2}} \gamma _i$ for all $i=1, \ldots , 8$, where $\gamma _i$ are the (“standard-normalization”) Gell–Mann matrices.

The (cross-)correlation matrix of ${\varvec{A}}, {\varvec{B}}$, denoted by ${\mathscr {C}}$, is defined by:

1 $$\begin{aligned} {\mathscr {C}}_{ij}&:=\left\langle A_i \otimes B_j \right\rangle = \mathrm {tr}\left( \rho A_i \otimes B_j\right) \end{aligned}$$

where $\rho$ is the density matrix shared by Alice and Bob.

As we shall see in the next section, the information contained in ${\mathscr {C}}$ regarding the strength of nonlocal correlations is encoded entirely in its singular values. An equivalent characterization is provided by a noteworthy relation between the singular value decomposition (SVD) of ${\mathscr {C}}$ and the *operator-Schmidt decomposition* of the underlying state, which we describe hereinafter. The operator-Schmidt decomposition of any state $\rho$ is defined as its unique decomposition of the form:

2 $$\begin{aligned} \rho = \sum _{k=1}^{d^2} \lambda _k G_k \otimes H_k \end{aligned}$$

where each $\lambda _k \ge 0$ is a real scalar, and the sets $\left\{ G_k \right\}$ and $\left\{ H_k \right\}$ are orthonormal sets of $d_{A} \times d_{A}, d_{B} \times d_{B}$ Hermitian matrices respectively. It can be shown that the singular values of ${\mathscr {C}}$ are precisely the Schmidt coefficients $\lambda _k$; moreover, the sets $\left\{ G_k \right\}$ and $\left\{ H_k \right\}$ are related to the sets $\left\{ A_i \right\}$ and $\left\{ B_j \right\}$ through the orthogonal matrices *U*, *V* of the SVD, respectively. Extended definitions and proof may be found in Section I.A of the supplementary information.

## Correlation minor norm

The goal of this work is to produce physical scalars from ${\mathscr {C}}$ that would allow for entanglement detection. In the context of this paper, a scalar is considered to be physical if it is invariant under a transformation of the set of measurements. Such a transformation is described by a pair of orthogonal matrices (see discussion in Section I.B of the supplementary information):

3 $$\begin{aligned} {\mathscr {C}} \rightarrow {\mathscr {U}}_A C {\mathscr {U}}_B^T , \qquad {\mathscr {U}}_{A} \in \mathrm {O} \left( d_{A}^2 \right) , {\mathscr {U}}_{B} \in \mathrm {O} \left( d_{B}^2 \right) . \end{aligned}$$

Introducing into (3) the SVD of ${\mathscr {C}}$, written as ${\mathscr {C}} = {\mathscr {V}}_A \Sigma \mathscr {V_B}^T$, yields:

4 $$\begin{aligned} {\mathscr {V}}_A \Sigma {\mathscr {V}}_B^T \rightarrow {\mathscr {U}}_A {\mathscr {V}}_A \Sigma {\mathscr {V}}_B^T {\mathscr {U}}_B^T . \end{aligned}$$

Since ${\mathscr {U}}_{A}$ and ${\mathscr {V}}_{A}$ are elements of $\mathrm {O} \left( d_{A}^2 \right)$ (and similarly for the matrices with the subscript *B*), we may observe that a general orthogonal transformation of ${\mathscr {C}} = {\mathscr {V}}_A \Sigma \mathscr {V_B}^T$ reduces to the substitution of ${\mathscr {V}}_A$ and ${\mathscr {V}}_B$ by any other elements of their respective orthogonal groups. Thus, it is clear that any physical scalar derived from ${\mathscr {C}}$ should depend on its singular values, i.e. the operator-Schmidt coefficients.

The simplest candidates for scalars produced by a matrix are its trace, determinant, and any type of matrix norm. However, $\mathrm {tr}\left( {\mathscr {C}}\right)$ is not a physical scalar in the sense described above; and a broad class of matrix norms are given as special cases of the scalars constructed in this section. Thus, for now we wish to consider $\det {\mathscr {C}}$ (where the discussion is restricted to $d_A = d_B$). The determinant of a quantum cross-correlation matrix can help detect entanglement, and may also serve as a measure of entanglement for two-qubit pure states (see Section I.D of the supplementary information) and two-mode Gaussian states^44–46^.

However, in more general scenarios, there are states in which the mutual information between Alice and Bob stems from specific subspaces of their respective vector spaces (in pure states, the dimension of these subspaces is given by the Schmidt rank). To accommodate these cases, one should go over all possible subspaces of some given dimension and consider the determinant of the matrix comprised of correlations between their basis elements. Then, one could construct a measure as some function of all those determinants. One way of doing so is treat them as entries of a matrix and take its *norm*.

In light of the observations above, we define the *Correlation Minor Norm with parameters* *h* *and* $p=2$:

5 $$\begin{aligned} {\mathscr {M}}_{h,p=2} :=\sqrt{ \sum _{R \in \left( {\begin{array}{c} \left[ d_A^2 \right] \\ h \end{array}}\right) } \sum _{S \in \left( {\begin{array}{c} \left[ d_B^2 \right] \\ h \end{array}}\right) } \left| \det {\mathscr {C}}_{R, S } \right| ^2 } \end{aligned}$$

where $$\left( {\begin{array}{c} \left[ a \right] \\ b\end{array}}\right)$$ denotes the set of *b*-combinations of $\left[ a\right]$ (this notation is common in the Cauchy–Binet formula), ${\mathscr {C}}_{R,S}$ is the matrix whose rows are the rows of ${\mathscr {C}}$ at indices from *R* and whose columns are the columns of ${\mathscr {C}}$ at indices from *S*, and $1 \le h \le \min \left\{ d_A^2, d_B^2 \right\}$. The meaning of the parameter *p* will become clear shortly, when the above definition is generalized.

Note that ${\mathscr {M}}_{h,2}$ is the Frobenius norm of a matrix ${\mathscr {N}}$ of size $$\left( {\begin{array}{c} d_A^2 \\ h \end{array}}\right) \times \left( {\begin{array}{c} d_B^2 \\ h \end{array}}\right)$$, defined by:

6 $$\begin{aligned} {\mathscr {N}}_{ij} \triangleq \det {\mathscr {C}}_{R_i, S_j} \end{aligned}$$

where we have numbered the sets’ elements:

$$\begin{aligned} \left( {\begin{array}{c} \left[ d_A^2 \right] \\ h \end{array}}\right) \triangleq \left\{ R_1, \ldots , R_{ \left( {\begin{array}{c} d_A^2 \\ h \end{array}}\right) } \right\} , \qquad \left( {\begin{array}{c} \left[ d_B^2 \right] \\ h \end{array}}\right) \triangleq \left\{ S_1, \ldots , S_{ \left( {\begin{array}{c} d_B^2 \\ h \end{array}}\right) } \right\} . \end{aligned}$$

Such a matrix ${\mathscr {N}}$ is known as the *h-th compound matrix of* ${\mathscr {C}}$, and is denoted by $C_h \left( {\mathscr {C}} \right)$. Now, recall the Schatten *p*-norm of any matrix *M* is defined by $\left|M\right|_p :=\left| \vec {\sigma } \left( M \right) \right|_p$, i.e. the *vector* *p*-norm of the vector composed of the singular values of *M*. Schatten *p*-norms lead to a generalization of the definition (5): for $p \in \left[ 1 , \infty \right)$, define the *Correlation Minor Norm with parameters* *h* *and* *p* as:

7 $$\begin{aligned} {\mathscr {M}}_{h,p} = \left| C_h \left( {\mathscr {C}} \right) \right|_p , \end{aligned}$$

i.e., it is the Schatten *p*-norm of the *h*-th compound matrix of the correlation matrix ${\mathscr {C}}$. Substituting the known relation between the singular values of any matrix and its compound matrix (see Section II of the supplementary information), one obtains the following formula for computing the Correlation Minor Norm (CMN):

8 $$\begin{aligned} {\mathscr {M}}_{h,p} = \left( \sum _{R \in \left( {\begin{array}{c} \left[ d^2 \right] \\ h\end{array}}\right) } \prod _{k \in R} \left[ \sigma _k \left( {\mathscr {C}} \right) \right] ^p \right) ^{1/p} , \end{aligned}$$

where $d = \min \left\{ d_A, d_B \right\}$, and $\sigma _k \left( {\mathscr {C}} \right)$ denotes the *k*-th singular value of ${\mathscr {C}}$. This implies that ${\mathscr {M}}_{h,p}$ is indeed a physical scalar. Note that the Schatten *p*-norm of ${\mathscr {C}}$ itself is obtained as a special case, for $h=1$. Another thing to note is that for $h = d^2$, the CMN ${\mathscr {M}}_{h,p}$ is equal to the product of all singular values, irregardless of *p*; in this case we denote it by ${\mathscr {M}}_{h=d^2}$. If $d_A=d_B$, this is simply $\det {\mathscr {C}}$.

## Entanglement detection using the correlation minor norm

For general mixed states, there are a few known links between Schmidt coefficients and entanglement detection; the best-known is probably the CCNR criterion: If $\sum _{k=1}^{d^2} \lambda _k > 1$, then $\rho$ is entangled^47^. The Correlation Minor Norm allows for an equivalent formulation: if ${\mathscr {M}}_{h=1,p=1} >1$, then $\rho$ is entangled.

The CCNR criterion has an additional immediate consequence regarding the CMN: since ${\mathscr {M}}_{h,p}$ is a monotonically increasing function of the operator-Schmidt coefficients $\lambda _k$, there is an upper bound for the value it may obtain without violating the inequality $\sum _{k=1}^{d^2} \lambda _k \le 1$. Thus, for all *h* and *p*, there exists some positive number $B = B \left( d_A, d_B, h, p \right)$ with the property: if $\rho$ is separable, then ${\mathscr {M}}_{h,p} \le B \left( d_A, d_B, h, p \right)$. This implies the Correlation Minor Norm can be used to detect entanglement by the following procedure: given a state $\rho$, the corresponding correlation matrix ${\mathscr {C}}$ is obtained - either by computation or by direct measurement; then, the SVD of ${\mathscr {C}}$ is used to find the singular values, and these are substituted in (8) to compute the desired CMN, ${\mathscr {M}}_{h,p}$; and finally, ${\mathscr {M}}_{h,p}$ is compared with $B \left( d_A, d_B, h, p \right)$. If ${\mathscr {M}}_{h,p} \le B \left( d_A, d_B, h, p \right)$, we cannot deduce anything. However, if ${\mathscr {M}}_{h,p} > B \left( d_A, d_B, h, p \right)$, we infer the state $\rho$ is entangled. The remainder of this section deals with results regarding the upper bounds $B \left( d_A, d_B, h, p \right)$. A technical treatment of the operator-Schmidt decomposition for separable states appears in Section III of the supplementary information.

In^40^, Lupo et al. generalize the CCNR criterion in the following way: they construct all *elementary symmetric polynomials* of the Schmidt coefficients $\lambda _k$ of $\rho$, and find bounds on these assuming $\rho$ is separable. The *h*-th elementary symmetric polynomial of *n* variables is defined as follows:

9 $$\begin{aligned} S_h \left( x_1, \ldots , x_n \right) :=\sum _{R \in \left( {\begin{array}{c}[n]\\ h\end{array}}\right) } \prod _{k \in R} x_k , \end{aligned}$$

i.e., the sum of all distinct products of *h* distinct variables. Clearly, ${\mathscr {M}}_{h,p=1} = S_h \left( \sigma _1, \ldots , \sigma _{d^2} \right)$.

A more recent work^41^ which cites^40^, makes the following important claim: assuming $d_A = d_B$, they find a tight bound on the *h*-th symmetric polynomial (for separable states), and prove that as an entanglement detector it is no stronger than the CCNR criterion. Since the conjectures presented in this section imply this is true for the CMN with $p = \infty$ as well, it seems likely that for $d_A = d_B$ and any value of *p*, the CMN is no stronger than the CCNR criterion as an entanglement detector.

However, in the case where $d_A \ne d_B$, it seems the CMN may detect entanglement in cases where CCNR does not. Let us define following^48–50^, *a state in Filter Normal Form (FNF)* as a state $\rho$ for which any traceless Alice-observable *A* and any traceless Bob-observable *B* have vanishing expectation values; i.e., $\left\langle A \otimes \mathbb {1}\right\rangle _\rho = \left\langle \mathbb {1} \otimes B\right\rangle _\rho = 0$. Then, we have the following result:

### Theorem 1

*Assume* $D :=\max \left\{ d_A, d_B \right\} \le d^3$ *and* $h >1$. *Then, for any separable state in Filter Normal Form:*

10 $$\begin{aligned} {\mathscr {M}}_{h,p=1} \le S_h \left( \alpha , \beta , \ldots , \beta \right) \end{aligned}$$

*where* $\alpha :=1/\sqrt{Dd}$, $\beta :=\sqrt{ \frac{D-1}{D \left( d^2-1\right) } \frac{d-1}{d \left( d^2-1\right) } }$, *and* $S_h$ *is the* *h*-*th elementary symmetric polynomial in* $d^2$ *variables.*

Proof may be found in Section IV.B of the supplementary information. Moreover, we conjecture the following theorem still holds with the assumption of the state being in FNF removed. If proven, this conjecture would have explained the upper bounds presented in^40^ for $d_A \ne d_B$, which had been found numerically.

Before presenting the next result, let us introduce quantum designs^51^. A *quantum design* in dimension *b* with *v* elements is simply a set of *v* orthogonal projections $\left\{ P_k \right\} _{k=1}^v$ on ${\mathbb {C}}^b$. A quantum design is *regular with* $r=1$ if all projections are pure (i.e. one-dimensional); it is *coherent* if the sum $\sum _k P_k$ is proportional to the identity operator; and it has *degree* 1 if there exists $\mu \in {\mathbb {R}}$ such that $\forall k \ne l, \mathrm {tr}\left( P_k P_l\right) = \mu$. If a quantum design has all three qualities, then $\mu = \frac{v -b }{b \left( v-1 \right) }$.

A regular, coherent, degree-1 quantum design with $r = 1$ having *v* elements, is simply a set of *v* “equally spaced” pure states in the same space. For example, such a quantum design in dimension *d* containing $d^2$ elements is known as a symmetric, informationally complete, positive operator-valued measure (SIC-POVM)^52^.

The following theorem tells us how to construct a separable state saturating (10) using quantum designs.

### Theorem 2

*Let* $\left\{ P_k^A \right\} _{k=1}^{d^2}$, $\left\{ P_k^B \right\} _{k=1}^{d^2}$ *be sets of pure projections comprising regular, coherent, degree-*1 *quantum designs with* $r = 1$, *in dimensions* $d_A, d_B$ *respectively and having* $d^2$ *elements each. Define a state:*

11 $$\begin{aligned} \rho = \frac{1}{d^2} \sum _{k=1}^{d^2} P_k^A \otimes P_k^B \end{aligned}$$

*Then, the operator-Schmidt coefficients of* $\rho$ *are* $\alpha$ *with multiplicity one and* $\beta$ *with multiplicity* $d^2-1$.

The proof appears in Section IV.C of the supplementary information. Note the last two theorems have the following special case: for $h=d^2$, they imply that the above state maximizes the product of all Schmidt coefficients; i.e., it maximizes ${\mathscr {M}}_{h=d^2,p}$ for all *p*.

Furthermore, we have similar claims for $p=\infty$.

### Theorem 3

*Let* $\rho$ *be a separable state in FNF, and* $h \ge \sqrt{Dd}$. *Then:*

12 $$\begin{aligned} {\mathscr {M}}_{h,p=\infty } \le \frac{1}{\sqrt{Dd}} \left[ \frac{D-1}{D \left( h-1\right) } \frac{d-1}{d \left( h-1\right) } \right] ^{\frac{h-1}{2}} . \end{aligned}$$

Proof may be found in Section IV.D of the supplementary information. As in Theorem 1, we conjecture this theorem still holds without the assumption that $\rho$ is in FNF. Evidence for why we believe this conjecture to be true may be found in Section IV.E of the supplementary information. The following theorem yields a way of saturating the bound (12):

### Theorem 4

*Let* $\left\{ P_k^A \right\} _{k=1}^{h}$, $\left\{ P_k^B \right\} _{k=1}^{h}$ *be sets of pure projections comprising regular, coherent, degree-*1 *quantum designs with* $r = 1$, *in dimensions* $d_A, d_B$ *respectively and having* *h* *elements each. Define a state:*

13 $$\begin{aligned} \rho = \frac{1}{h} \sum _{k=1}^{h} P_k^A \otimes P_k^B \end{aligned}$$

*Then, the operator-Schmidt coefficients of* $\rho$ *are* $\alpha$ *with multiplicity one and* $\beta ' = \sqrt{ \frac{D-1}{D \left( h-1\right) } \frac{d-1}{d \left( h-1\right) } }$ *with multiplicity* $h-1$.

The proof appears in in Section IV.F of the supplementary information. Note the coherence of $\left\{ P_k^{A/B} \right\}$ ensures the state (13) is in FNF. Moreover, the constants $\mu _{A/B} :=\frac{h -d_{A/B} }{d_{A/B} \left( h-1 \right) }$ enter the operator-Schmidt coefficients (and thus the upper bound (12)) elegantly: $\beta ' = \sqrt{ \frac{1-\mu _A}{h} \frac{1-\mu _B}{h} }$.

We hypothesize that upper bounds over ${\mathscr {M}}_{h,p}$ for any value of *p* may be characterized using quantum designs. If this hypothesis is proven, then separable states built using such quantum designs are, in a way, on the “edges” of the convex separable set. However, one should note that quantum designs in a given dimension with a given number of elements do not always exist; the above theorems hold only in the cases where they do exist.

## Further results and examples

### Relation to entanglement entropy for pure states

Let $\left| \psi \right\rangle$ be a pure state, and let $s_1,\ldots ,s_d$ denote its “pure-state-Schmidt coefficients” (i.e., the ones arising when writing the Schmidt decomposition for pure states of $\left| \psi \right\rangle$). Then, its operator-Schmidt coefficients are $s_k s_l$, i.e. all the pairwise products of pure-state-Schmidt coefficients (if $k \ne l$, $s_k s_l$ appears as an operator-Schmidt coefficient with multiplicity 2; for proof please refer to in Section I.C of the supplementary information.

**Figure 1:**
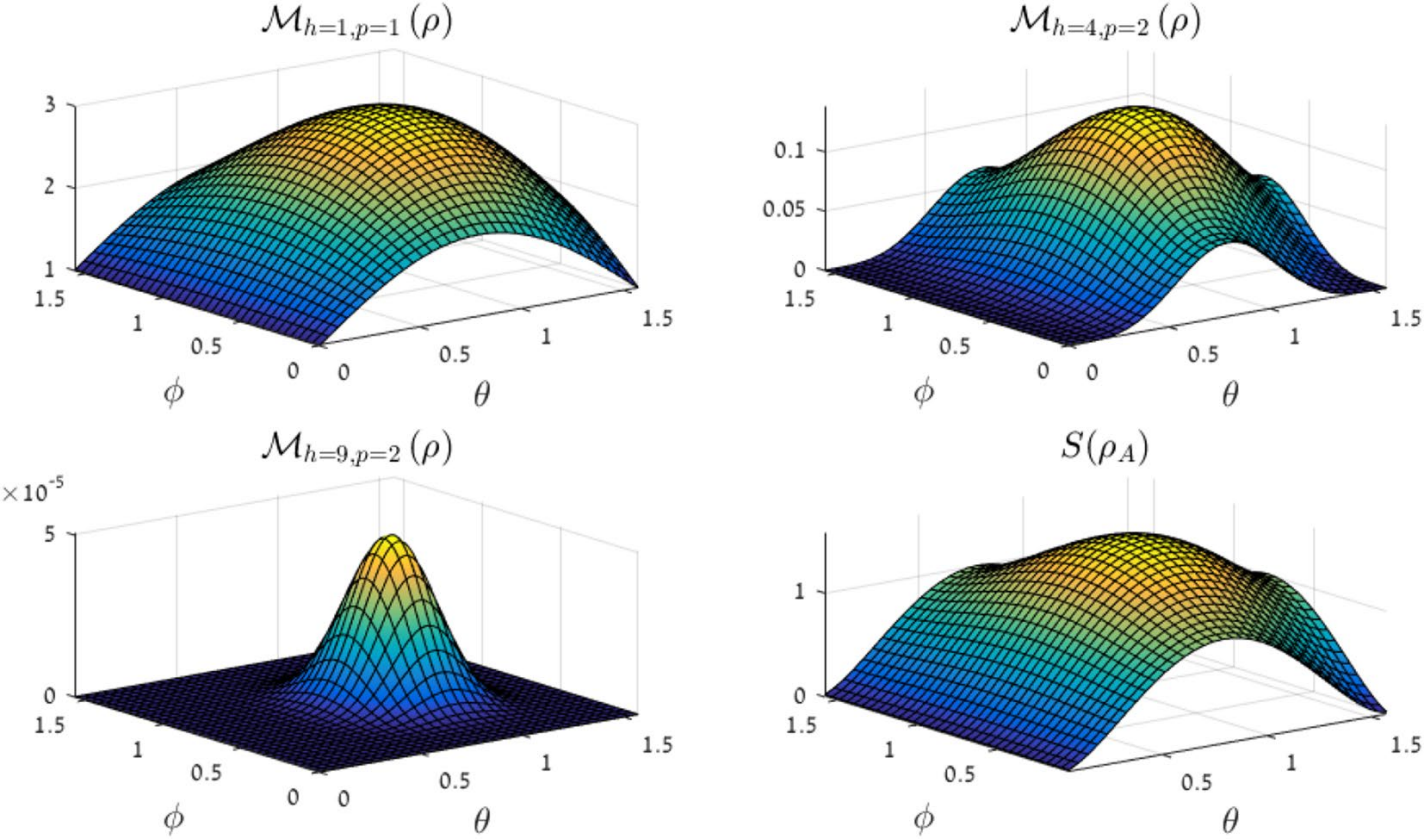
Several CMNs ${\mathscr {M}}_{h,p}$ and the von-Neumann entanglement entropy $S \left( \rho _A \right)$, plotted for the two-qutrit states with pure-state-Schmidt coefficients given in spherical coordinates: $s_1 = \sin \theta \cos \phi$, $s_2 = \sin \theta \sin \phi$ and $s_3 = \cos \theta$ (to avoid repetition, only the domain $0 \le \theta , \pi \le \pi /2$ is plotted). The area where $S \left( \rho _A \right)$ vanishes, $\theta = 0, \pi / 2$, alludes to the domain where the state’s Schmidt rank is 1, and the same goes for ${\mathscr {M}}_{h=2^2,p}$. However, ${\mathscr {M}}_{h=3^2,p}$ also tells us where the Schmidt rank is 2, i.e., $\phi = 0, \pi /2$. (This figure was created using MATLAB R2016A).

For pure states, the Correlation Minor Norm is linked to the state’s Schmidt rank by the following observation: for all $t \in \left[ d \right]$, ${\mathscr {M}}_{h=t^2,p} \ne 0$ iff the state’s pure-state-Schmidt rank is at least *t*. Thus, the Correlation Minor Norm may be used to find the Schmidt rank in pure states. Figure 1 illustrates a comparison between ${\mathscr {M}}_{h=t^2, p}$ and entanglement entropy for all two-qutrit pure states.

Moreover, for any pure state of dimension $2 \times D$, the CMN and entanglement entropy are only functions of $s_1^2$, and both functions have the same monotonicity w.r.t. this parameter (i.e. they increase / decrease in the same domains). This may be demonstrated by noting that effectively, the qubit is only correlated with a two-dimensional subsystem of Bob’s system. Using the same reasoning that appears in Section I.D of the supplementary information, it could be argued that ${\mathscr {M}}_{h=4}$ may indeed quantify entanglement in this scenario.

However, it is clear that not all Correlation Minor Norms are useful for this purpose; in fact, the relation between operator-Schmidt coefficients and pure-state-Schmidt coefficients implies:

14 $$\begin{aligned} {\mathscr {M}}_{h=1,p=2}^2 = \sum _k \lambda _k^2 = \sum _{k,l} \left( s_k s_l \right) ^2 = \left( \sum _k s_k^2 \right) ^2 = 1 . \end{aligned}$$

Thus, ${\mathscr {M}}_{h=1,p=2} = 1$ for *any* pure state, be it separable or entangled.

### Improving on the CCNR criterion

In this section, we shall present an entangled state which may be detected by the CMN, but *cannot* be detected by the CCNR criterion. First, let $\rho _0$ be the state (11) for $d_A = 3, d_B = 2$; and let $\rho _1 :=\left| \psi \right\rangle \left\langle \psi \right|$, where $\psi :=\left( \left| 11\right\rangle + \left| 20\right\rangle \right) /\sqrt{2}$. The state is constructed as follows:

15 $$\begin{aligned} \rho _q = q \rho _1 + \left( 1-q\right) \rho _0 . \end{aligned}$$

For $q=0.295$ the state is entangled (easily verifiable by the PPT criterion). However, it is not detected by the CCNR criterion: ${\mathscr {M}}_{h=1,p=1} = 0.9981 < 1$; and it is detected by the CMN: ${\mathscr {M}}_{h=2,p=1} = 0.3509$, exceeding the bound $\frac{2+ 3 \sqrt{2} }{18} \approx 0.3468$.

### Relation to quantum discord

Since the CMN seems to capture some value related to quantum correlations, it is intriguing to ask whether it may somehow be be used to measure their strength. The geometric measure for quantum discord (GQD) with respect to Alice’s subsystem is defined^38^ as:

16 $$\begin{aligned} {\mathscr {D}}_G^A \left( \rho \right) :=\min _{\chi \in c-q} \left| \rho - \chi \right|^2 , \end{aligned}$$

i.e. the shortest squared Euclidean distance between $\rho$ and any classical-quantum state (the expression for discord w.r.t. Bob’s subsystem is defined similarly, where the minimization goes over all quantum-classical states).

Motivated by this definition and by the expression for GQD derived in^39^, we suggest the following measure for discord w.r.t. Alice’s subsystem, based on the CMN:

17 $$\begin{aligned} {\mathscr {D}}^A_{h,p} \left( \rho \right) = \left[ {\mathscr {M}}_{h,p} \left( \rho \right) \right] ^p - \max _{\Pi ^A \in M \left( A \right) } \left[ {\mathscr {M}}_{h,p} \left( \Pi ^A \left[ \rho \right] \right) \right] ^p , \end{aligned}$$

where the maximization goes over all projective measurements on Alice’s subsystem $\Pi ^A = \left\{ \Pi _i \right\} _{i=1}^{d_A}$, and $\Pi ^A \left[ \rho \right]$ is the state obtained from $\rho$ by performing the measurement $\Pi ^A$ and obtaining the appropriate ensemble of the projections $\Pi _i$ (i.e., the state is measured but not “collapsed”). The following result suggests that ${\mathscr {D}}^A_{h,p}$ may be thought of as a measure for discord:

#### Theorem 5

*For any state* $\rho$ *and for any value of* *h*, *p*, ${\mathscr {D}}^A_{h,p} \left( \rho \right) \ge 0$; *and* ${\mathscr {D}}^A_{h\le 2,p} \left( \rho \right) = 0$ *iff* ${\mathscr {D}}_G^A \left( \rho \right) = 0$.

Moreover, for any state $\rho$, we have ${\mathscr {D}}_G^A \left( \rho \right) = {\mathscr {D}}^A_{h=1,p=2}$. The proof for this fact, as well as for the theorem above, appears in Section V of the supplementary information. As evident in the proof, ${\mathscr {D}}_G^A \left( \rho \right) = 0 \; \Rightarrow \; {\mathscr {D}}^A_{h,p} \left( \rho \right) = 0$ for $h>2$ as well.

**Figure 2:**
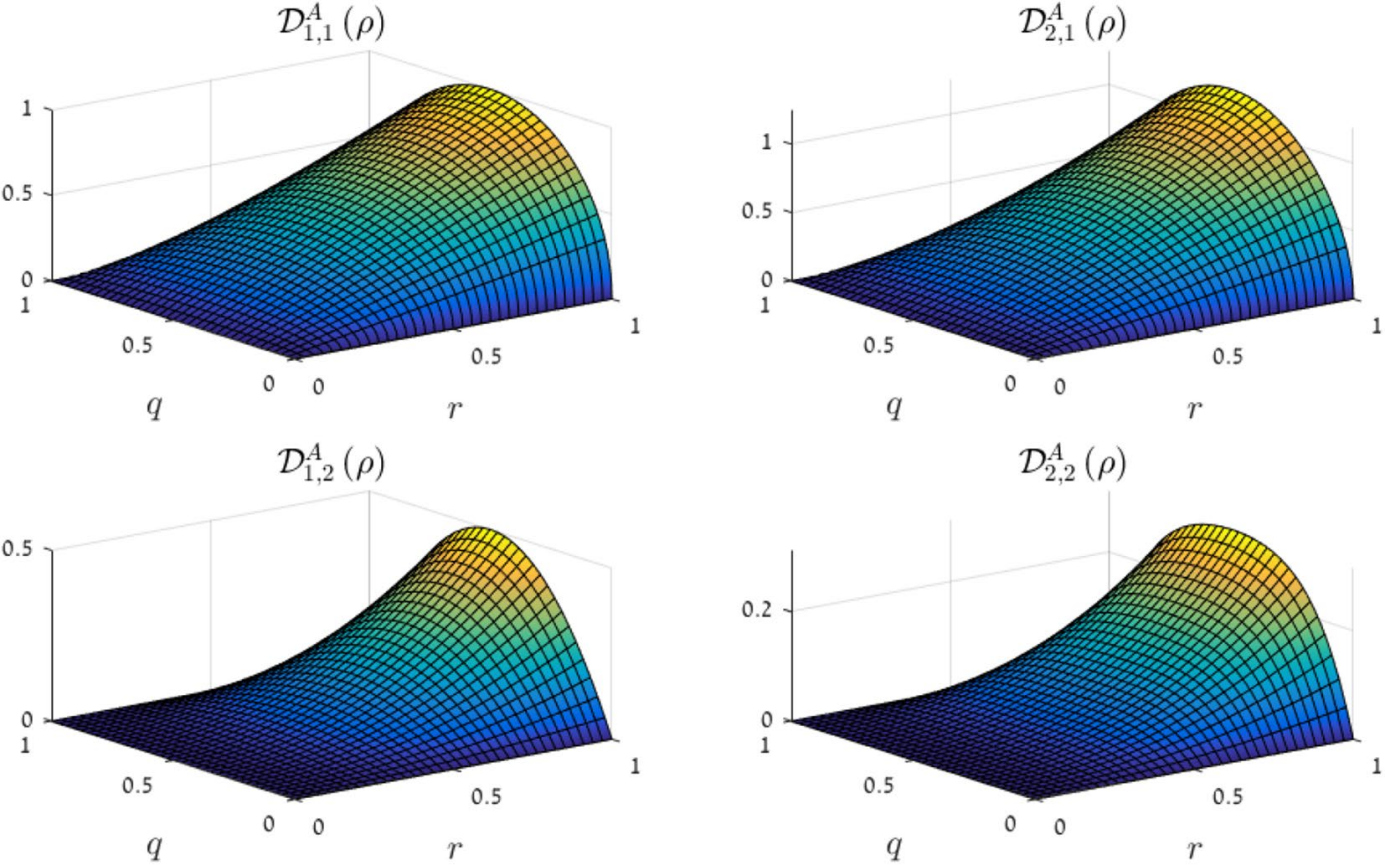
The CMN-motivated measures for geometric quantum discord ${\mathscr {D}}_{h,p}^A$, for the family of states given in^53^, with parameters $q,r \in \left[ 0, 1 \right]$. The discord is computed using the formula given on^53^. The similarity is not a coincidence, as the geometric discord of these states is given by a product of two operator-Schmidt coefficients (times a factor of 2). (This figure was created using MATLAB R2016A).

Figure 2 illustrates several of the measures ${\mathscr {D}}^A_{h,p} \left( \rho \right)$ for a two-parameter family of states given in^53^. The states appear in Section V of the supplementary information. It is also worth noting in this context, that the two-qubit separable state with maximal discord has the same operator-Schmidt coefficients as the Werner state with $c=1/3$^37,54^ (hence they are unitarily equivalent); and it is precisely the state maximizing the CMNs with $p=1$. In other words—the entanglement, discord and CMNs for the two-qubit Werner states are all monotonically increasing functions of the parameter $c \in \left[ 0, 1 \right]$, and for the critical value of *c* (above which the states are entangled) the Werner state is precisely the one for which the CMN obtains its separable upper bound. This situation occurs for the two-qutrit Werner state as well, where the critical value of *c* is 1/4 (when constructed as in^55^).

## Conclusions

The task of entanglement detection is important for basic quantum science, as well as various quantum technologies. The current work was motivated by the following question: since bipartite entanglement can be characterized by correlations between all of the parties’ observables, can it also be detected via some norm of these correlations? As demonstrated by our results, the answer is likely to be affirmative.

We have defined the Correlation Minor Norm and explored its characteristics. This has allowed us to propose an approach for detecting entanglement both in pure and mixed states. Furthermore, it was shown that for pure states, the Correlation Minor Norm allows one to determine the Schmidt rank, and in some cases also quantify the strength of quantum correlations. Given the dimensions of the two parties’ respective systems, one may choose a single set of operators which can be used for detecting entanglement in any state, be it pure or mixed.

Additionally, we have shown that the CMN with $h=1,p=2$ admits a natural relation to geometric quantum discord. This affinity motivated a definition of a more general measure for quantum discord which is based on the CMN. Some of these measures might mitigate the known issues with existing discord measures^56–59^.

One optional direction for future research may include development of dynamical equations for the Correlation Minor Norm. This may be interesting, as the correlation matrix contains exactly the same information as the density matrix.

Another possible generalization is considering multipartite systems. In^60^, the authors consider detection of genuine multipartite entanglement and non-full-separability using correlation tensors. Specifically, they consider tensors comprising all multipartite correlations between orthonormal bases to the *traceless* observables; and they find upper bounds on norms of *matricizations* of these tensors, such that exceeding these bounds implies the state is genuine multipartite entangled, or non-fully-separable.

This paper may hint as to how our work may be generalized to the multipartite case: one could consider the *full* correlation tensor (i.e. correlations between bases to the entire space of observables, not just the traceless ones); then, the CMN with parameters *h*, *p* may be defined as the Schatten *p*-norm of the *h*th compound matrix of a certain matricization of this tensor. The bounds shown in^60^ could then be utilized to find two upper bounds on each of the CMNs—one for non-genuinely-entangled states, and another for fully-separable states. The question of which matricization should be used remains to be determined. Moreover, further work is required to find the states saturating these bounds.

## Supplementary Information

Supplementary Information.

## References

[1] Islam R (2015). Measuring entanglement entropy in a quantum many-body system. Nature.

[2] Amico L, Fazio R, Osterloh A, Vedral V (2008). Entanglement in many-body systems. Rev. Mod. Phys..

[3] Jurcevic P (2014). Quasiparticle engineering and entanglement propagation in a quantum many-body system. Nature.

[4] Kaufman AM (2016). Quantum thermalization through entanglement in an isolated many-body system. Science.

[5] Braunstein SL, Van Loock P (2005). Quantum information with continuous variables. Rev. Mod. Phys..

[6] Bello, L., Michael, Y., Rosenbluh, M., Cohen, E. & Pe’er, A. Complex two-mode quadratures—a unified formalism for continuous-variable quantum optics. *arXiv preprint* arXiv:2011.08099 (2020).

[7] Berrada K, Abdel-Khalek S (2011). Entanglement of atom-field interaction for nonlinear optical fields. Phys. E Low Dimens. Syst. Nanostruct..

[8] Abdel-Khalek S, Berrada K, Ooi CR (2012). Beam splitter entangler for nonlinear bosonic fields. Laser Phys..

[9] Chtchelkatchev NM, Blatter G, Lesovik GB, Martin T (2002). Bell inequalities and entanglement in solid-state devices. Phys. Rev. B.

[10] Wieśniak M, Vedral V, Brukner Č (2005). Magnetic susceptibility as a macroscopic entanglement witness. New J. Phys..

[11] González-Tudela A, Porras D (2013). Mesoscopic entanglement induced by spontaneous emission in solid-state quantum optics. Phys. Rev. Lett..

[12] Tichy MC, Mintert F, Buchleitner A (2011). Essential entanglement for atomic and molecular physics. J. Phys. B.

[13] Sackett CA (2000). Experimental entanglement of four particles. Nature.

[14] Jaksch D, Briegel H-J, Cirac J, Gardiner C, Zoller P (1999). Entanglement of atoms via cold controlled collisions. Phys. Rev. Lett..

[15] Yönaç M, Yu T, Eberly J (2006). Sudden death of entanglement of two Jaynes-cummings atoms. J. Phys. B.

[16] Berrada K, Fanchini FF, Abdel-Khalek S (2012). Quantum correlations between each qubit in a two-atom system and the environment in terms of interatomic distance. Phys. Rev. A.

[17] Mohamed A-B, Eleuch H, Ooi CR (2019). Non-locality correlation in two driven qubits inside an open coherent cavity: Trace norm distance and maximum bell function. Sci. Rep..

[18] Peres A (1996). Separability criterion for density matrices. Phys. Rev. Lett..

[19] Horodecki M, Horodecki P, Horodecki R (1996). Separability of mixed states: Necessary and sufficient conditions. Phys. Lett. A.

[20] Horodecki P (1997). Separability criterion and inseparable mixed states with positive partial transposition. Phys. Lett. A.

[21] Brandao FG (2005). Quantifying entanglement with witness operators. Phys. Rev. A.

[22] Gühne O (2004). Characterizing entanglement via uncertainty relations. Phys. Rev. Lett..

[23] Gühne O, Hyllus P, Gittsovich O, Eisert J (2007). Covariance matrices and the separability problem. Phys. Rev. Lett..

[24] Gühne O, Mechler M, Tóth G, Adam P (2006). Entanglement criteria based on local uncertainty relations are strictly stronger than the computable cross norm criterion. Phys. Rev. A.

[25] Gittsovich O, Gühne O, Hyllus P, Eisert J (2008). Unifying several separability conditions using the covariance matrix criterion. Phys. Rev. A.

[26] Li J-L, Qiao C-F (2018). A necessary and sufficient criterion for the separability of quantum state. Sci. Rep..

[27] de Vicente JI (2007). Separability criteria based on the Bloch representation of density matrices. Quantum Inf. Comput..

[28] Carmi A, Cohen E (2018). On the significance of the quantum mechanical covariance matrix. Entropy.

[29] Carmi A, Cohen E (2019). Relativistic independence bounds nonlocality. Sci. Adv..

[30] Te’eni A, Peled BY, Cohen E, Carmi A (2019). Multiplicative bell inequalities. Phys. Rev. A.

[31] Pozsgay V, Hirsch F, Branciard C, Brunner N (2017). Covariance bell inequalities. Phys. Rev. A.

[32] Ollivier H, Zurek WH (2001). Quantum discord: A measure of the quantumness of correlations. Phys. Rev. Lett..

[33] Zurek WH (2000). Einselection and decoherence from an information theory perspective. Ann. Phys..

[34] Henderson L, Vedral V (2001). Classical, quantum and total correlations. J. Phys. A.

[35] Giorda P, Paris MG (2010). Gaussian quantum discord. Phys. Rev. Lett..

[36] Luo S (2008). Quantum discord for two-qubit systems. Phys. Rev. A.

[37] Bera A (2017). Quantum discord and its allies: A review of recent progress. Rep. Prog. Phys..

[38] Dakić B, Vedral V, Brukner Č (2010). Necessary and sufficient condition for nonzero quantum discord. Phys. Rev. Lett..

[39] Luo S, Fu S (2010). Geometric measure of quantum discord. Phys. Rev. A.

[40] Lupo C, Aniello P, Scardicchio A (2008). Bipartite quantum systems: On the realignment criterion and beyond. J. Phys. A.

[41] Li C-K, Poon Y-T, Sze N-S (2011). A note on the realignment criterion. J. Phys. A.

[42] Chen K, Wu L-A (2002). A matrix realignment method for recognizing entanglement. Quantum Inf. Comput..

[43] Rudolph O (2005). Further results on the cross norm criterion for separability. Quantum Inf. Process..

[44] Simon R (2000). Peres–Horodecki separability criterion for continuous variable systems. Phys. Rev. Lett..

[45] Dodonov A, Dodonov V, Mizrahi S (2004). Separability dynamics of two-mode gaussian states in parametric conversion and amplification. J. Phys. A.

[46] De Castro A, Dodonov V (2006). Purity and squeezing exchange between coupled bosonic modes. Phys. Rev. A.

[47] Gühne O, Tóth G (2009). Entanglement detection. Phys. Rep..

[48] Kent A, Linden N, Massar S (1999). Optimal entanglement enhancement for mixed states. Phys. Rev. Lett..

[49] Verstraete F, Dehaene J, De Moor B (2003). Normal forms and entanglement measures for multipartite quantum states. Phys. Rev. A.

[50] Leinaas JM, Myrheim J, Ovrum E (2006). Geometrical aspects of entanglement. Phys. Rev. A.

[51] Zauner G (2011). Quantum designs: Foundations of a noncommutative design theory. Int. J. Quantum Inf..

[52] Renes JM, Blume-Kohout R, Scott AJ, Caves CM (2004). Symmetric informationally complete quantum measurements. J. Math. Phys..

[53] Virzì S (2019). Optimal estimation of entanglement and discord in two-qubit states. Sci. Rep..

[54] Galve F, Giorgi GL, Zambrini R (2011). Maximally discordant mixed states of two qubits. Phys. Rev. A.

[55] Ye B, Liu Y, Chen J, Liu X, Zhang Z (2013). Analytic expressions of quantum correlations in qutrit Werner states. Quantum Inf. Process..

[56] Piani M (2012). Problem with geometric discord. Phys. Rev. A.

[57] Tufarelli T, Girolami D, Vasile R, Bose S, Adesso G (2012). Quantum resources for hybrid communication via qubit-oscillator states. Phys. Rev. A.

[58] Paula F, de Oliveira TR, Sarandy M (2013). Geometric quantum discord through the Schatten 1-norm. Phys. Rev. A.

[59] Roga W, Spehner D, Illuminati F (2016). Geometric measures of quantum correlations: Characterization, quantification, and comparison by distances and operations. J. Phys. A.

[60] de Vicente JI, Huber M (2011). Multipartite entanglement detection from correlation tensors. Phys. Rev. A.

