# Supplementary material for: Correlation minor norms, entanglement detection and discord
Source: Sci Rep. 2021 Feb 2;11:2849. doi: 10.1038/s41598-021-82303-3 (PMC7854613; doi:10.1038/s41598-021-82303-3)
Supplement: Supplementary file 1 — Supplementary Information. [file 41598_2021_82303_MOESM1_ESM.pdf]

Supplementary information for "Correlation Minor Norm as a Detector and Quantifier of Entanglement"

Bar Y. Peled,[1] Amit Te'eni,[2] Avishy Carmi,[1] and Eliahu Cohen[2]

[1] *Center for Quantum Information Science and Technology & Faculty of Engineering Sciences, Ben-Gurion University of the Negev, Beersheba 8410501, Israel*

[2] *Faculty of Engineering and the Institute of Nanotechnology and Advanced Materials, Bar Ilan University, Ramat Gan 5290002, Israel*

I. ENTANGLEMENT DETECTION USING THE QUANTUM CORRELATION MATRIX

A. The Operator-Schmidt Decomposition

Given any state ρ (either separable or entangled), one may write down the following unique decomposition:

$$\rho = \sum_{k=1}^{d^2} \lambda_k G_k \otimes H_k \tag{1}$$

where $d := \min\{d_A, d_B\}$, each $\lambda_k \geq 0$ is a real scalar, and the sets $\{G_k\}$ and $\{H_k\}$ form orthonormal bases of the $d_{A/B} \times d_{A/B}$ Hermitian matrices. Note this is not necessarily a "separable decomposition", since G_k, H_k are not compelled to be positive semi-definite.

Let us assume that λ_k are in non-increasing order. We shall demonstrate that the SVD of the cross-correlation matrix $\mathcal{C}$ is equivalent to the Operator-Schmidt Decomposition.

Theorem. *Given a state ρ, let $\mathcal{C}$ be the second moment matrix of the orthonormal sets $\{A_i\}, \{B_j\}$, defined by $\mathcal{C}_{ij} = \langle A_i \otimes B_j \rangle_\rho$. Let $\mathcal{C}$ have the SVD $\mathcal{C} = U\Sigma V^T$ with singular values $\sigma_1 \geq \ldots \geq \sigma_{d^2}$. Then, the unique decomposition (1) of ρ satisfies the following:*

1. $\lambda_k = \sigma_k$
2. $G_k = \sum_{i=1}^{d^2} U_{ik} A_i$
3. $H_k = \sum_{j=1}^{d^2} V_{jk} B_j$.

Proof outline: since $\{A_i \otimes B_j\}_{i,j}$ comprise a basis to the set of $d^2 \otimes d^2$ Hermitian matrices, the matrix $\mathcal{C}$ suffices in order to fully characterize ρ. Thus, since the Operator-Schmidt decomposition is unique, all is left to do is verify that ρ with the above Operator-Schmidt decomposition reproduces the same correlations $\mathcal{C}_{ij}$, which is straightforward.

B. Change of Measurement Basis

Mathematically, Alice and Bob's observables transform by the representation $\boldsymbol{d^2} = \boldsymbol{d^2 - 1} \oplus \boldsymbol{1}$ (adjoint plus trivial singlet) of a local projective unitary transformation $U \in \mathrm{PU}(d)$ (where d is either d_A or d_B):

$$A_i \to U_A A_i U_A^\dagger, \ B_j \to U_B B_j U_B^\dagger, \tag{2}$$

where we have taken the projective unitary groups $\mathrm{PU}(d) = \mathrm{PSU}(d) = \mathrm{U}(d)/\mathrm{U}(1) = \mathrm{SU}(d)/\mathbb{Z}_d$, since any $\mathrm{U}(1)$ phase clearly cancels out in the above. The trivial part is given by the identity component of A_i (or B_j), and the adjoint part by the traceless component. Since the adjoint representation of $\mathrm{PU}(d)$ is a subgroup of $\mathrm{SO}(d^2-1)$, there exists a basis where the vector of observables $[A_i]$ transforms by:

$$\begin{pmatrix} A_1 \\ \vdots \\ A_{d_A^2} \end{pmatrix} \to \begin{bmatrix} 1 & 0 \\ 0 & R \end{bmatrix} \begin{pmatrix} A_1 \\ \vdots \\ A_{d_A^2} \end{pmatrix}, \tag{3}$$

where A_1 is a scalar matrix, and $R \in \mathrm{SO}(d_A^2 - 1)$. For instance, suppose $d_A = d_B = 2$; then, Alice's observables may correspond to measurements of a spin-1/2 in a given orthonormal set of directions. If the first measurement is fixed to be the trivial one $\mathbb{1}/\sqrt{2}$, then a special orthogonal transformation $R \in \mathrm{SO}(3)$ describes a rotation of Alice's entire lab; similarly, Bob's lab may be rotated independently of Alice's.

However, if the first measurement is not fixed, then we should consider more general transformations than those with the form (3). Since the required basis transformation preserves inner the Hilbert-Schmidt inner product, it can be taken to be a $d \times d$ orthogonal matrix; indeed, the matrices of the form (3) are naturally embedded in $\mathrm{O}(d_A^2)$. Therefore, the correlation matrix furnishes a tensor product of two representations (i.e. Alice's and Bob's), described by

$$\mathcal{C} \to \mathcal{U}_A \mathcal{C} \mathcal{U}_B^T, \qquad \mathcal{U}_A \in \mathrm{O}(d_A^2), \mathcal{U}_B \in \mathrm{O}(d_B^2). \tag{4}$$

C. Operator-Schmidt decomposition for pure states

Let $|\psi\rangle$ be a pure state given in its pure-state-Schmidt decomposition:

$$|\psi\rangle = \sum_{k=1}^{d} s_k |\phi_k\rangle \otimes |\xi_k\rangle . \tag{5}$$

The appropriate density matrix:

$$\rho = |\psi\rangle\langle\psi| = \sum_{k,l=1}^{d} s_k s_l |\phi_k\rangle\langle\phi_l| \otimes |\xi_k\rangle\langle\xi_l|. \quad (6)$$

Let us fix k, l s.t. $k < l$. k and l appear in two terms of the sum: $s_k s_l (|\phi_k\rangle\langle\phi_l| \otimes |\xi_k\rangle\langle\xi_l| + |\phi_l\rangle\langle\phi_k| \otimes |\xi_l\rangle\langle\xi_k|)$. We wish to write down the parenthesized expression in the form $G_{kl} \otimes H_{kl} + G_{lk} \otimes H_{lk}$, where $G_{kl}, H_{kl}, G_{lk}, H_{lk}$ are all trace-normalized Hermitian operators, and $\mathrm{tr}(G_{kl}G_{lk}) = \mathrm{tr}(H_{kl}H_{lk}) = 0$. Indeed, this is achieved by setting:

$$\begin{aligned} G_{kl} &:= \frac{|\phi_k\rangle\langle\phi_l| + |\phi_l\rangle\langle\phi_k|}{\sqrt{2}}; \\ H_{kl} &:= \frac{|\xi_k\rangle\langle\xi_l| + |\xi_l\rangle\langle\xi_k|}{\sqrt{2}}; \\ G_{lk} &:= \frac{i(|\phi_k\rangle\langle\phi_l| - |\phi_l\rangle\langle\phi_k|)}{\sqrt{2}}; \\ H_{lk} &:= -\frac{i(|\xi_k\rangle\langle\xi_l| - |\xi_l\rangle\langle\xi_k|)}{\sqrt{2}}. \end{aligned} \quad (7)$$

By supplementing the notations $G_{kk} := |\phi_k\rangle\langle\phi_k|$, $H_{kk} := |\xi_k\rangle\langle\xi_k|$, one may write (6) by:

$$\rho = \sum_{k,l=1}^{d} s_k s_l G_{kl} \otimes H_{kl}. \quad (8)$$

Since $\{G_{kl}\}$ and $\{H_{kl}\}$ are both orthonormal sets of operators, (8) is the operator-Schmidt decomposition of ρ; thus, $\{s_k s_l\}$ are its operator-Schmidt coefficients.

D. $\det \mathcal{C}$ for two-qubit pure states

Let $|\psi\rangle$ be a two-qubit pure state, given in its pure-state-Schmidt decomposition:

$$|\psi\rangle = s_1 |\phi_1\rangle \otimes |\xi_1\rangle + s_2 |\phi_2\rangle \otimes |\xi_2\rangle. \quad (9)$$

From the previous subsection, its operator-Schmidt coefficients are $s_1^2, s_1 s_2, s_2 s_1, s_2^2$. Moreover, from Section I A of this supplemental material, these are also the singular values of its correlation matrix. Thus:

$$\det \mathcal{C} = \prod_k \sigma_k(\mathcal{C}) = s_1^4 s_2^4 = \left(s_1^2 s_2^2\right)^2 = \left[s_1^2\left(1 - s_1^2\right)\right]^2, \quad (10)$$

where the final transition follows from the normalization condition $s_1^2 + s_2^2 = 1$. An interesting observation is that $\sqrt[4]{\det \mathcal{C}}$ is proportional to the interferometric distinguishability measure studied in [1–5]; moreover, [2] illustrates the striking resemblance between this measure and entangelement entropy. Thus, for two-qubit pure states, $\det \mathcal{C}$ indeed quantifies entanglement. As an aside, we note that the distinguishability measure is generalized for a certain family of Gaussian states in [6].

II. CMN AND SVD

In order to compute the CMN, one should seek a relation between the singular values of given matrix, and the singular values of its compound matrices. Such a relation is known [7]:

Lemma. *Let E be a $n \times n$ matrix. The singular values of $C_h(E)$, are the $\binom{n}{h}$ possible products $\sigma_{i_1} \cdots \sigma_{i_h}$.*

Which implies:

$$\|C_h(E)\|_p = \left(\sum_{R \in \binom{[n]}{h}} \prod_{k \in R} [\sigma_k(E)]^p\right)^{1/p} \quad (11)$$

where:

$$\binom{[n]}{h} \triangleq \left\{R \in 2^{[n]} : |R| = h\right\} \quad (12)$$

i.e., $\binom{[n]}{h}$ denotes the set of subsets of $[n]$ having cardinality h. Thus we obtain the following formula for the correlation minor norm, using only the singular values of the second moment matrix:

$$\mathcal{M}_{h,p} = \left(\sum_{R \in \binom{[d^2]}{h}} \prod_{k \in R} [\sigma_k(\mathcal{C})]^p\right)^{1/p}. \quad (13)$$

Note that the CMN yields another formulation for the CCNR criterion:

$$\forall \rho \in \mathcal{S}, \quad \mathcal{M}_{h=1,p=1} \leq 1, \quad (14)$$

where $\mathcal{S}$ denotes the set of separable states. The CMN also allows for a new formulation of the CM criterion [8]: For any separable state in FNF, $\mathcal{M}_{h=1,p=1} \leq \frac{1+\sqrt{(D-1)(d-1)}}{\sqrt{Dd}}$. Note the RHS is strictly smaller than 1 iff $D \neq d$.

III. THE OPERATOR-SCHMIDT DECOMPOSITION OF A SEPARABLE STATE

Assume $D = d_A \geq d_B = d$. We wish to find the Schmidt coefficients of the following density matrix:

$$\rho = \sum_{k=1}^{n} p_k O_k \otimes Q_k. \quad (15)$$

A. Aside: $n = d^2$

First, let us prove we can always assume that $n = d^2$ (however, O_k, Q_k are not necessarily pure): Suppose $n > d^2$. It suffices to show we can always transform (15) to a

similar state with $n-1$. Since the Q_k all belong to the space of $d\times d$ Hermitian matrices, they must be linearly dependent; i.e., thus, one of them (w.l.g. it is Q_n) may be written as a linear combination of the others:

$$\exists c_1,\ldots,c_{n-1}: Q_n=\sum_{k=1}^{n-1}c_kQ_k \tag{16}$$

where $\mathrm{tr}\,(Q_n)=1$ implies $\sum c_k=1$. Plugging this into (15) yields:

$$\begin{aligned}\rho&=\sum_{k=1}^{n-1}p_kO_k\otimes Q_k+p_nO_n\otimes\sum_{k=1}^{n-1}c_kQ_k=\\&=\sum_{k=1}^{n-1}\left(p_kO_k+p_nc_kO_n\right)\otimes Q_k=\\&=\sum_{k=1}^{n-1}\underbrace{(p_k+p_nc_k)}_{\tilde{p}_k}\underbrace{\frac{p_kO_k+p_nc_kO_n}{p_k+p_nc_k}}_{\tilde{O}_k}\otimes Q_k.\end{aligned}\tag{17}$$

To conclude the proof, one should verify $\sum_{k=1}^{n-1}\tilde{p}_k=1$ and $\mathrm{tr}\left(\tilde{O}_k\right)=1$. This is straightforward so we do not show it here.

B. Realignment and correlation in Bloch vector representation

Let us write the realigned density matrix:

$$\rho_R=\sum_{k=1}^{n}p_k\,\mathrm{vec}\,O_k\,\mathrm{vec}\,Q_k{}^\dagger. \tag{18}$$

Now, we shall write down $\rho_R^\dagger\rho_R$ as a "superoperator" $\hat{\mathscr{P}}$ - i.e., its operates on $d\times d$ Hermitian operators:

$$\hat{\mathscr{P}}=\sum_{k=1}^{n}p_kO_k\otimes Q_k \tag{19}$$

here the tensor product sign $\otimes$ has a meaning closer to its original one, rather than its regular abuse in quantum information theory; that is, it "wants" to act on a $d\times d$ Hermitian operator with the Hilbert-Schmidt inner product as follows:

$$\left(A\otimes B\right)C=\left\langle B,C\right\rangle A=\mathrm{tr}\left(B^\dagger C\right)A \tag{20}$$

where B,C are both all $d\times d$ (Hermitian) operators. For the sake of simplicity, we switch to the Bloch representation of the operators, satisfying the following properties:

1. Each operator Q_k is written as $Q_k=\frac{1}{\sqrt{d}}q_k^\mu\hat{\sigma}_\mu$, where $\mu=0,1,\ldots,d^2-1$. $\hat{\sigma}_0=\mathbb{1}_d/\sqrt{d}$, and the other $\hat{\sigma}_i$ are (traceless) $d\times d$ Hermitian operators s.t. all $\hat{\sigma}_\mu$ are an orthogonal set (w.r.t. the Hilbert-Schmidt inner product), satisfying:
$$\mathrm{tr}\left(\hat{\sigma}_\nu\hat{\sigma}_\mu\right)=\delta_{\nu\mu}$$
and the q_k^μ are real numbers, given by:
$$q_k^\mu=\sqrt{d}\cdot\mathrm{tr}\left(\hat{\sigma}_\mu Q_k\right). \tag{21}$$
Note that $q_k^0=\mathrm{tr}\left(Q_k\right)=1$.

2. This notation allows one to compute the Hilbert-Schmidt inner product of two operators $U=\frac{1}{\sqrt{d}}u^\nu\hat{\sigma}_\nu$ and $V=\frac{1}{\sqrt{d}}v^\mu\hat{\sigma}_\mu$ as follows:
$$\begin{aligned}\left\langle U,V\right\rangle_{HS}=&\mathrm{tr}\left(UV\right)=\frac{1}{d}\mathrm{tr}\left(u^\nu\hat{\sigma}_\nu v^\mu\hat{\sigma}_\mu\right)=\\&=\frac{1}{d}u^\nu v^\mu\mathrm{tr}\left(\hat{\sigma}_\nu\hat{\sigma}_\mu\right)=\frac{1}{d}u^\mu v_\mu.\end{aligned}$$

And similarly for the operators O_k (where d is replaced with $D:=\max\{d_A,d_B\}=d_A$):

$$O_k=\frac{1}{\sqrt{D}}o_k^\gamma\hat{\xi}_\gamma,\quad \mathrm{tr}\left(\hat{\xi}_\gamma\hat{\xi}_\eta\right)=\delta_{\gamma\eta},\quad o_k^\gamma=\sqrt{D}\cdot\mathrm{tr}\left(\hat{\xi}_\gamma O_k\right). \tag{22}$$

Once Hermitian operators are represented by column vectors (using the bases $\left\{\hat{\xi}_\gamma\right\},\left\{\hat{\sigma}_\mu\right\}$), the superoperator $\hat{\mathscr{P}}$ may once again be written as a $D^2\times d^2$ matrix:

$$\mathcal{C}^{\gamma\mu}=\frac{1}{\sqrt{Dd}}\sum_{k=1}^{n}p_ko_k^\gamma q_k^\mu=\frac{1}{\sqrt{Dd}}\mathcal{O}\mathcal{P}\mathcal{Q}^T, \tag{23}$$

where $\mathcal{O}^\gamma{}_l:=o_l^\gamma,\mathcal{Q}^\mu{}_k:=q_k^\mu$ are the matrices with columns comprised of the Bloch vectors of O_l,Q_k respectively; and $\mathcal{P}:=\mathrm{diag}\left[p_1,\ldots,p_n\right]$. Later on, it shall be useful to consider the matrix $\mathcal{R}:=\mathcal{C}^T\mathcal{C}$, since its eigenvalues are the squared singular values of $\mathcal{C}$:

$$\mathcal{R}=\frac{1}{Dd}\left(\mathcal{O}\mathcal{P}\mathcal{Q}^T\right)^T\mathcal{O}\mathcal{P}\mathcal{Q}^T=\frac{1}{Dd}\mathcal{Q}\mathcal{P}\mathcal{O}^T\mathcal{O}\mathcal{P}\mathcal{Q}^T. \tag{24}$$

C. Separability

Up until this point we still haven't used the *separability* of ρ; it manifests in the fact that the operators O_k,Q_l all represent states, implying:

$$\forall k,\begin{cases}\mathrm{tr}\left(O_k\right)=\mathrm{tr}\left(Q_k\right)=1 &\Rightarrow\quad o_k^0=q_k^0=1\\ \mathrm{tr}\left(O_k^2\right)\le1\wedge\mathrm{tr}\left(Q_k^2\right)\le1 &\Rightarrow o_k^\mu o_k^\mu\le D,\,q_k^\nu q_k^\nu\le d,\end{cases} \tag{25}$$

where only the Greek indices are summed upon. I.e., the first row of $\mathcal{O},\mathcal{Q}$ is all ones; and the main diagonals of $\frac{1}{D}\mathcal{O}^T\mathcal{O}$, $\frac{1}{d}\mathcal{Q}^T\mathcal{Q}$ are bounded by one. Let us denote $\mathcal{O}_+,\mathcal{Q}_+$ as the matrices obtained by removing the all-ones first rows from $\mathcal{O},\mathcal{Q}$ respectively. It would be useful to unify the two conditions, by writing down the summa-

tion explicitly:

$$\forall k, \quad 1 \geq \operatorname{tr}\left(O_k^2\right) = \frac{1}{D}\sum_{\mu=0}^{D^2-1} o_k^{\mu} o_k^{\mu} = \\ = \frac{1}{D}\left(1 + \sum_{\mu=1}^{D^2-1} o_k^{\mu} o_k^{\mu}\right) = \frac{1}{D}\left(1 + \left[\mathcal{O}_+^T \mathcal{O}_+\right]_{kk}\right), \tag{26}$$

implying:

$$\forall k, \quad \left[\mathcal{O}_+^T \mathcal{O}_+\right]_{kk} \leq D - 1 \tag{27}$$

and similarly:

$$\forall k, \quad \left[\mathcal{Q}_+^T \mathcal{Q}_+\right]_{kk} \leq d - 1. \tag{28}$$

Finally, we note that:

$$r^{\gamma} = \mathcal{C}^{\gamma 0} = \frac{1}{\sqrt{Dd}} \sum_k p_k o_k^{\gamma} \tag{29}$$

implying:

$$\boldsymbol{r} = \frac{1}{\sqrt{Dd}} \mathcal{O}_+ \boldsymbol{p}. \tag{30}$$

This is unsurprising, since $\left[1, \boldsymbol{r}^T\right]$ is the Bloch vector of $\rho_A = \sum_k p_k O_k$. Similarly:

$$\boldsymbol{s} = \frac{1}{\sqrt{Dd}} \mathcal{Q}_+ \boldsymbol{p}. \tag{31}$$

D. FNF

Let us assume that Alice and Bob choose their orthonormal observables such that $A_1 = \frac{1}{\sqrt{d_A}} \mathbb{1}_{d_A}$ and $B_1 = \frac{1}{\sqrt{d_B}} \mathbb{1}_{d_B}$, i.e. the trivial measurements. Note this implies that all the other observables A_i, B_j are traceless. Given this assumption, we are motivated to introduce the following notation (similar to [9]):

$$\mathcal{C} = \begin{bmatrix} 1/\sqrt{Dd} & \boldsymbol{s}^T \\ \boldsymbol{r} & \mathcal{T} \end{bmatrix}, \tag{32}$$

i.e.: $r_i := \langle A_i \otimes \mathbb{1} \rangle / \sqrt{d_B}, s_j := \langle \mathbb{1} \otimes B_j \rangle / \sqrt{d_A}$, and $\mathcal{T}$ is the correlation matrix of only traceless observables. A state ρ is said to be in FNF if $\boldsymbol{r} = \boldsymbol{0}$ and $\boldsymbol{s} = \boldsymbol{0}$. Any state may be transformed to FNF (using SLOCC), such that the original state is separable iff the transformed state is separable.

We note:

$$\mathcal{T} = \frac{1}{\sqrt{dD}} \mathcal{O}_+ \mathcal{P} {\mathcal{Q}_+}^T. \tag{33}$$

A recent paper [10] has used similar ideas to construct a necessary and sufficient separability criterion; in fact, they state that a correlation matrix $\mathcal{T}$ describes a separable state in FNF iff it admits a decomposition of the form (33), where $P = \operatorname{diag} \boldsymbol{p}$ is diagonal, real, non-negative and has unit trace; and $\mathcal{O}_+ \boldsymbol{p} = \boldsymbol{0}$, $\mathcal{Q}_+ \boldsymbol{p} = \boldsymbol{0}$. The latter conditions are related to the FNF: from (15), we observe that ρ is in FNF iff $\sum_k p_k O_k, \sum_k p_k Q_k$ are both proportional to the identity; considering this statement in Bloch vector terms readily implies these conditions.

IV. PROVING THE UPPER BOUNDS

This section uses the notation and results detailed in Section III of this supplemental material to prove the main results of our work.

A. Preliminaries

Observe the following:

$$\sum_{j=1}^{d^2-1} \sigma_j\left(\mathcal{T}\right) = \frac{1}{\sqrt{Dd}} \sum_{k=1}^{d^2-1} \sigma_k\left(\mathcal{O}_+ \mathcal{P} {\mathcal{Q}_+}^T\right) = \\ = \frac{1}{\sqrt{Dd}} \left\| \mathcal{O}_+ \sqrt{\mathcal{P}} \sqrt{\mathcal{P}} \mathcal{Q}_+^T \right\|_1 \leq \frac{1}{\sqrt{Dd}} \left\| \mathcal{O}_+ \sqrt{\mathcal{P}} \right\|_2 \left\| \mathcal{Q}_+ \sqrt{\mathcal{P}} \right\|_2 \tag{34}$$

where the final transition follows from Hölder's inequality. Let us find bounds on the 2-norms:

$$\left\| \mathcal{O}_+ \sqrt{\mathcal{P}} \right\|_2^2 = \operatorname{tr}\left(\sqrt{\mathcal{P}} \mathcal{O}_+^T \mathcal{O}_+ \sqrt{\mathcal{P}}\right) = \operatorname{tr}\left(\mathcal{P} \mathcal{O}_+^T \mathcal{O}_+\right) = \\ = \sum_{k=1}^{d^2} p_k \left[\mathcal{O}_+^T \mathcal{O}_+\right]_{kk} \leq D - 1 \tag{35}$$

and similarly,

$$\left\| \mathcal{Q}_+ \sqrt{\mathcal{P}} \right\|_2^2 \leq d - 1. \tag{36}$$

Substitution of the latter two in (34) yields

$$\sum_{k=1}^{d^2-1} \sigma_k \leq \sqrt{\frac{D-1}{D} \frac{d-1}{d}}. \tag{37}$$

Note this inequality is equivalent to the separability criterion defined by de Vicente in [9] (the dV criterion). de Vicente defines a matrix T similar to our $\mathcal{T}$; in fact, $T = \frac{Dd}{2} \mathcal{T}$. The dV criterion states that for any separable state,

$$\|T\|_{KF} \leq \sqrt{\frac{Dd\left(D-1\right)\left(d-1\right)}{4}}, \tag{38}$$

where $\|\cdot\|_{KF}$ denotes the Ky-Fan norm, i.e. the Schatten 1-norm (also known as the trace norm or nuclear norm).

(38) implies:

$$\sum_{j=1}^{d^2-1} \sigma_j(\mathcal{T}) = \|\mathcal{T}\|_{KF} = \frac{2}{Dd}\|T\|_{KF} \leq \sqrt{\frac{D-1}{D}\frac{d-1}{d}}. \quad (39)$$

B. Proof of the upper bound of $\mathcal{M}_{h,p=1}$

In this subsection, we wish to use the results of the previous section to find bounds on $\mathcal{M}_{h,p=1} = \|C_h(\mathcal{C})\|_1$ for separable states. Clearly, the tight upper bound for $h=1$ is $\mathcal{M}_{h=1,p=1} \leq 1$ (CCNR). Here we add three other assumptions: one regarding the domain of h - $h>1$; another is $D \leq d^3$; and finally, we assume the state is in FNF.

Thus, $\sigma_0 = 1/\sqrt{Dd}$, and we obtain:

$$\mathcal{M}_{h,p=1} = S_h\left(1/\sqrt{Dd}, \sigma_1, \ldots, \sigma_{d^2-1}\right) = \\ = \frac{1}{\sqrt{Dd}} S_{h-1}(\sigma_1, \ldots, \sigma_{d^2-1}) + S_h(\sigma_1, \ldots, \sigma_{d^2-1}) \quad (40)$$

Let us denote $s := \sum_{k=1}^{d^2-1}\sigma_k$ and $\beta := \frac{1}{d^2-1}\sqrt{\frac{D-1}{D}\frac{d-1}{d}}$. Clearly $s \leq \beta\left(d^2-1\right)$. Moreover, the vectors $\vec{\sigma} := (\sigma_1, \ldots, \sigma_{d^2-1})$ and $\vec{e} := \frac{s}{d^2-1}(1, \ldots, 1)$ both sum up to s; thus, $\vec{\sigma} \succeq \vec{e}$ ($\succeq$ denotes majorization). Since the symmetric polynomials S_h are Schur concave, we obtain:

$$S_h(\sigma_1, \ldots, \sigma_{d^2-1}) \leq S_h\left(\frac{s}{d^2-1}, \ldots, \frac{s}{d^2-1}\right). \quad (41)$$

Next, we use the fact that S_h is monotonically increasing in each of its variables, alongside the inequality $\frac{s}{d^2-1} \leq \beta$, to obtain:

$$S_h(\sigma_1, \ldots, \sigma_{d^2-1}) \leq S_h(\beta, \ldots, \beta). \quad (42)$$

Substitution in (40) yields:

$$\mathcal{M}_{h,p=1} \leq \alpha S_{h-1}(\beta, \ldots, \beta) + S_h(\beta, \ldots, \beta) = \\ = S_h(\alpha, \beta, \ldots, \beta) \quad (43)$$

where β is always repeated d^2-1 times.

C. Saturating the upper bound of $\mathcal{M}_{h,p=1}$

In our special construction of ρ from Theorem 2 in the paper, $n=d^2$ and $\forall k, p_k = 1/d^2$. The following additional assumptions follow from $\{O_k\}, \{Q_l\}$ being regular, coherent, degree-1 quantum designs with $r=1$ and d^2 elements:

$$\frac{1}{D}\left[\mathcal{O}^T\mathcal{O}\right]_{kl} = \langle O_k, O_l\rangle = \begin{cases} 1; & k=l \\ \mu_A; & k \neq l \end{cases}, \\ \frac{1}{d}\left[\mathcal{Q}^T\mathcal{Q}\right]_{kl} = \langle Q_k, Q_l\rangle = \begin{cases} 1; & k=l \\ \mu_B; & k \neq l \end{cases} \quad (44)$$

where $\mu_{A/B} = \frac{d^2-d_{A/B}}{d_{A/B}(d^2-1)}$. Coherence has the following additional implication:

$$\sum_{k=1}^{d^2} O_k = \frac{d^2}{D}\mathbb{1}_D, \quad \sum_{k=1}^{d^2} Q_k = d\mathbb{1}_d \\ \Rightarrow \sum_{k=1}^{d^2} o_k^\mu = \sum_{k=1}^{d^2} q_k^\mu = \begin{cases} d^2; & \mu=0 \\ 0; & \mu \neq 0 \end{cases} \quad (45)$$

in matrix notation:

$$\mathcal{O}\mathbf{1} = \mathcal{Q}\mathbf{1} = \begin{bmatrix} d^2 \\ 0 \\ \vdots \\ 0 \end{bmatrix}, \quad \mathcal{O}_+\mathbf{1} = \mathcal{Q}_+\mathbf{1} = \mathbf{0}. \quad (46)$$

where $\mathbf{1}$ is the vector whose d^2 entries all equal 1. Furthermore, we know that such a quantum design in dimension $d = d_B$ is in fact a SIC-POVMs; thus, $\{Q_k\}$ are SIC-POVMs.

Substituting these implications allows one to obtain:

$$\mathcal{R}_{\mu\nu} = \frac{1}{d^5}\sum_{k=1}^{d^2} q_k^\mu q_k^\nu + \frac{\mu_A}{d^5}\sum_{k\neq l} q_k^\mu q_l^\nu. \quad (47)$$

Moreover, we have:

$$\mathcal{R}_{00} = \frac{1}{d^5}\sum_{k,l=1}^{d^2}\langle O_k, O_l\rangle \underbrace{q_k^0 q_l^0}_{=1} = \frac{1}{d^5}\langle\sum_{k=1}^{d^2} O_k, \sum_{l=1}^{d^2} O_l\rangle = \\ = \frac{1}{D^2 d}\langle\mathbb{1}_D, \mathbb{1}_D\rangle = \frac{1}{d_A d_B}. \quad (48)$$

and for all $\nu \neq 0$:

$$\mathcal{R}_{0\nu} = \frac{1}{d^5}\sum_{k,l=1}^{d^2}\langle O_k, O_l\rangle \underbrace{q_k^0}_{=1} q_l^\nu = \frac{1}{d^5}\sum_{l=1}^{d^2}\langle\sum_{k=1}^{d^2} O_k, O_l\rangle q_l^\nu = \\ = \frac{1}{Dd^3}\sum_{l=1}^{d^2}\underbrace{\langle\mathbb{1}_D, O_l\rangle}_{\operatorname{tr}(O_l)=1} q_l^\nu = 0. \quad (49)$$

Similarly, for all $\mu \neq 0$, $\mathcal{R}_{\mu 0} = 0$. Thus, $\lambda_0 = \mathcal{R}_{00} = \frac{1}{d_A d_B}$ is an eigenvalue. To conclude the proof, we just need to show that the submatrix of $\mathcal{R}$ without the first row and column - i.e., $\mathcal{T}^T\mathcal{T}$ - is the scalar matrix $\beta^2\mathbb{1}$.

To do so, we note the following:

$$\mathcal{T}^T\mathcal{T} = \frac{1}{Dd^5}\mathcal{Q}_+\mathcal{O}_+^T\mathcal{O}_+\mathcal{Q}_+^T \quad (50)$$

where we have plugged $\mathcal{P} = \frac{\mathbb{1}}{d^2}$ into (33). Our first step would be computing $\mathcal{O}_+^T\mathcal{O}_+$. Using (44) and recalling that $\mathcal{O}_+$ is simply $\mathcal{O}$ with the first row of all 1s removed, we obtain:

$$\left[\mathcal{O}_+^T\mathcal{O}_+\right]_{kl} = \left[\mathcal{O}^T\mathcal{O}\right]_{kl} - 1 = \begin{cases} D-1; & k=l \\ -\frac{D-1}{d^2-1}; & k \neq l \end{cases} \quad (51)$$

Diagonalization of this matrix is rather straightforward; it is not difficult to obtain that it has two distinct eigenvalues:

1. $\lambda_0 =$ with multiplicity 1, where the eigenspace is spanned by $\mathbf{1} := (1, \ldots, 1)^T$; and -
2. $\lambda_1 = \frac{d^2(D-1)}{d^2-1}$ with multiplicity $d^2 - 1$ and eigenspace $\Lambda := (\mathrm{span}\{\mathbf{1}\})^\perp$.

This demonstrates that $\mathcal{O}_+^T \mathcal{O}_+$ behaves as a scalar matrix, when its domain is restricted to $\Lambda \subset \mathbb{R}^{d^2}$. Thus, our next step would be showing that $\mathcal{Q}_+^T : \mathbb{R}^{d^2-1} \to \mathbb{R}^{d^2}$ performs exactly this restriction.

In other words, we wish to prove that $\mathrm{Im}\, \mathcal{Q}_+^T = \Lambda$. First note $\mathcal{Q}_+^T$ has an empty kernel, since otherwise there exists a nonzero vector orthogonal to all vectors in $\{\boldsymbol{q}_k^+\}$; this would have implied that $\{\boldsymbol{q}_k^+\}$ do not span the entire d^2-1-dimensional space of traceless Hermitian $d \times d$ operators, contradicting them comprising a SIC-POVM. Thus $\ker \mathcal{Q}_+^T = 0$, and from the rank-nullity theorem $\mathcal{Q}_+^T$ must have rank $d^2 - 1$. Hence, demonstrating that $\mathrm{Im}\, \mathcal{Q}_+^T \subset \Lambda$ would complete the proof. Let $\boldsymbol{v} \in \mathbb{R}^{d^2-1}$; indeed, direct computation yields:

$$\mathbf{1} \cdot \mathcal{Q}_+^T \boldsymbol{v} = \mathcal{Q}_+ \mathbf{1} \cdot \boldsymbol{v} = \mathbf{0}, \tag{52}$$

where we have used (46). Thus, for all $\boldsymbol{v} \in \mathbb{R}^{d^2-1}$ we have:

$$\mathcal{O}_+^T \mathcal{O}_+ \mathcal{Q}_+^T \boldsymbol{v} = \frac{d^2 (D-1)}{d^2 - 1} \mathcal{Q}_+^T \boldsymbol{v}, \tag{53}$$

implying,

$$\mathcal{O}_+^T \mathcal{O}_+ \mathcal{Q}_+^T = \frac{d^2 (D-1)}{d^2 - 1} \mathcal{Q}_+^T. \tag{54}$$

To complete the proof, we note that since $\{Q_k\}$ comprises a SIC-POVM, the columns of $\mathcal{Q}_+$ form a (real) equiangular tight frame in dimension $d^2 - 1$ with d^2 elements [11]; thus, its *frame operator* $\mathcal{Q}_+ \mathcal{Q}_+^T$ is scalar; more specifically, it satisfies:

$$\mathcal{Q}_+ \mathcal{Q}_+^T = A \mathbb{1}_{d^2-1}, \tag{55}$$

where A is readily found by taking the trace of both sides:

$$\left(d^2 - 1\right) A = \mathrm{tr}\left(\mathcal{Q}_+ \mathcal{Q}_+^T\right) = \mathrm{tr}\left(\mathcal{Q}_+^T \mathcal{Q}_+\right) = d^2 (d-1), \tag{56}$$

where we have used (44) again. Multiplying (54) by $\mathcal{Q}_+$ from the left yields:

$$\begin{aligned}\mathcal{Q}_+ \mathcal{O}_+^T \mathcal{O}_+ \mathcal{Q}_+^T &= \frac{d^2 (D-1)}{d^2 - 1} \mathcal{Q}_+ \mathcal{Q}_+^T = \\ &= \frac{d^2 (D-1)}{d^2 - 1} \frac{d^2 (d-1)}{d^2 - 1} \mathbb{1}_{d^2-1},\end{aligned} \tag{57}$$

which, when plugged into (50), concludes the proof.

D. Proof of the upper bound of $\mathcal{M}_{h,p=\infty}$

In this subsection we prove the bound on $\mathcal{M}_{h,p=\infty} = \|C_h(\mathcal{C})\|_\infty$ for separable states in FNF. Clearly, the tight upper bound for $h = 1$ is $\mathcal{M}_{h=1,p=\infty} \leq 1$.

Thus, $\sigma_0 = 1/\sqrt{Dd}$, and we obtain:

$$\begin{aligned}\mathcal{M}_{h,p=\infty} &= \|C_h(\mathcal{C})\|_\infty = \frac{1}{\sqrt{Dd}} \|C_{h-1}(\mathcal{T})\|_\infty = \\ &= \frac{1}{\sqrt{Dd}} \left\| C_{h-1}\left(\frac{1}{\sqrt{Dd}} \mathcal{O}_+ \mathcal{P} {\mathcal{Q}_+}^T\right)\right\|_\infty = \\ &= (Dd)^{-h/2} \prod_{k=1}^{h-1} \sigma_k\left(\mathcal{O}_+ \mathcal{P} {\mathcal{Q}_+}^T\right) \leq \\ &\leq (Dd)^{-h/2} (h-1)^{-(h-1)} \left[\sum_{k=1}^{h-1} \sigma_k\left(\mathcal{O}_+ \mathcal{P} {\mathcal{Q}_+}^T\right)\right]^{h-1}.\end{aligned} \tag{58}$$

Let us find a bound on the sum:

$$\begin{aligned}\sum_{k=1}^{h-1} \sigma_k\left(\mathcal{O}_+ \mathcal{P} {\mathcal{Q}_+}^T\right) &\leq \sum_{k=1}^{d^2} \sigma_k\left(\mathcal{O}_+ \mathcal{P} {\mathcal{Q}_+}^T\right) \leq \\ &\leq \sqrt{(D-1)(d-1)}\end{aligned} \tag{59}$$

where we have used (37). To conclude, substituting in (58) obtains the bound:

$$\mathcal{M}_{h,p=\infty} \leq \frac{1}{\sqrt{Dd}} \left[\frac{D-1}{D(h-1)} \frac{d-1}{d(h-1)}\right]^{\frac{h-1}{2}}. \tag{60}$$

E. Evidence to support Theorem 3 from the paper without assuming FNF

$\mathcal{M}_{h,p=\infty}$ is a monotonically non-decreasing differentiable function of the singular values $\vec{\sigma} = (\sigma_0, \ldots, \sigma_{d^2-1})$. The constraints on the domain of $\vec{\sigma}$ are rather complicated and we do not know them all. However, we know *some* of them:

$$\sigma_0 \geq 1/\sqrt{Dd} \tag{61}$$

$$\forall j \in \left\{1, \ldots, d^2 - 1\right\}, \quad \sigma_j \geq 0 \tag{62}$$

$$\sum_{j=0}^{d^2} \sigma_j \leq 1 \tag{63}$$

$$\sum_{j=1}^{d^2-1} \sigma_j \leq \frac{D-1}{D} \frac{d-1}{d}. \tag{64}$$

Furthermore, we know from numerical simulations that (63) and (64) cannot be saturated simultaneously (for $Dd \leq h^2$); in fact, it seems that if the latter is saturated, then the state must be in FNF (thus saturating (61) instead). *Assume* that this statement holds in general, and that no other constraints on $\vec{\sigma}$ are relevant for

global maxima analysis of $\mathcal{M}_{h,p=\infty}$ - i.e., no other constraints need be saturated to obtain its global maxima; then, the theorem holds.

Since $\mathcal{M}_{h,p=\infty}$ is monotonically increasing, one of the constraints (63),(64) must be saturated in a global maximum; otherwise, any one of the σ_k could be increased, thus increasing the value of $\mathcal{M}_{h,p=\infty}$ without leaving the domain. According to our assumption, if (64) is saturated the state is in FNF, which is the case we already treated. Thus, assume (63) is saturated. If more than h singular values are nonzero, the point cannot be a global maximum, since we can increase the largest singular value while decreasing the smallest nonzero singular value, thus leaving (63) saturated while increasing $\mathcal{M}_{h,p=\infty}$. Thus, we may treat $\mathcal{M}_{h,p=\infty}$ as a function depending only on the h largest singular values:

$$f(\vec{\sigma}) = \prod_{k=0}^{h-1} \sigma_k, \tag{65}$$

And we are currently considering a global maximum $\vec{\sigma}' = (\sigma_0, \ldots, \sigma_{h-1})$ s.t. $\sum_{k=0}^{h-1} \sigma_k = 1$. Clearly, non of the σ_k can be zero - otherwise $\vec{\sigma}'$ is a minimum rather than a maximum. Thus, of all the above constraints, $\vec{\sigma}'$ saturates only (63). Thus, it should be a *local* maximum of the following function constructed using a Lagrange multiplier:

$$g(\vec{\sigma}, \lambda) := \prod_{k=0}^{h-1} \sigma_k - \lambda \left(\sum_{k=0}^{h-1} \sigma_k - 1 \right). \tag{66}$$

Thus, the partial derivatives with respect to σ_k should vanish:

$$0 = \frac{\partial g}{\partial \sigma_l} = \prod_{k \neq l} \sigma_k - \lambda \tag{67}$$

implying that for all l, $\prod_{k \neq l} \sigma_k = \lambda$; but that could only happen if $\sigma_0 = \ldots = \sigma_{h-1} = 1/h$. Substituting $\sigma_0 = 1/h$ in (61) would have implied $h \leq \sqrt{Dd}$. If this is an equality, we are again in FNF; otherwise, it contradicts one of our initial assumptions. Thus, the only possible global maximum is the one obtained in FNF, for which Theorem 3 from our paper holds.

F. Saturating the upper bound of $\mathcal{M}_{h,p=\infty}$

In our special construction of ρ from Theorem 4 in the paper, $n = h$ and $\forall k, p_k = 1/h$. Since $\{O_k\}, \{Q_l\}$ are regular, coherent, degree-1 quantum designs with $r = 1$ and h elements, (44) still holds; the only difference is that in this case, $\mu_{A/B} = \frac{h - d_{A/B}}{d_{A/B}(h-1)}$. As before, coherence has an additional implication:

$$\sum_{k=1}^{h} O_k = \frac{h}{D} \mathbb{1}_D, \quad \sum_{k=1}^{h} Q_k = \frac{h}{d} \mathbb{1}_d$$
$$\Rightarrow \quad \sum_{k=1}^{h} o_k^{\mu} = \sum_{k=1}^{h} q_k^{\mu} = \begin{cases} h; & \mu = 0 \\ 0; & \mu \neq 0 \end{cases} \tag{68}$$

in matrix notation:

$$\mathcal{O}\mathbf{1} = \mathcal{Q}\mathbf{1} = \begin{bmatrix} h \\ 0 \\ \vdots \\ 0 \end{bmatrix}, \qquad \mathcal{O}_+\mathbf{1} = \mathcal{Q}_+\mathbf{1} = \mathbf{0}, \tag{69}$$

where $\mathbf{1}$ is the vector whose h entries all equal 1.

Substituting these implications allows one to obtain:

$$\mathcal{R}_{\mu\nu} = \frac{1}{h^2 d} \sum_{k=1}^{h} q_k^{\mu} q_k^{\nu} + \frac{\mu_A}{h^2 d} \sum_{k \neq l} q_k^{\mu} q_l^{\nu}. \tag{70}$$

Moreover, we have:

$$\mathcal{R}_{00} = \frac{1}{h^2 d} \sum_{k,l=1}^{h} \langle O_k, O_l \rangle \underbrace{q_k^0 q_l^0}_{=1} = \frac{1}{h^2 d} \langle \sum_{k=1}^{h} O_k, \sum_{l=1}^{h} O_l \rangle =$$
$$= \frac{1}{D^2 d} \langle \mathbb{1}_D, \mathbb{1}_D \rangle = \frac{1}{d_A d_B}. \tag{71}$$

and for all $\nu \neq 0$:

$$\mathcal{R}_{0\nu} = \frac{1}{h^2 d} \sum_{k,l=1}^{h} \langle O_k, O_l \rangle \underbrace{q_k^0}_{=1} q_l^{\nu} =$$
$$= \frac{1}{h^2 d} \sum_{l=1}^{h} \langle \sum_{k=1}^{h} O_k, O_l \rangle q_l^{\nu} = \frac{1}{hDd} \sum_{l=1}^{h} \underbrace{\langle \mathbb{1}_D, O_l \rangle}_{\mathrm{tr}(O_l)=1} q_l^{\nu} = 0. \tag{72}$$

Similarly, for all $\mu \neq 0$, $\mathcal{R}_{\mu 0} = 0$. Thus, $\lambda_0 = \mathcal{R}_{00} = \frac{1}{d_A d_B}$ is an eigenvalue. To conclude the proof, we need to show that the submatrix of $\mathcal{R}$ without the first row and column - again, $\mathcal{T}^T\mathcal{T}$ - is composed of two diagonal blocks, one being a nontrivial scalar matrix and the other is the zero matrix.

We commence in a manner similar to what we have done in subsection IV C - writing down $\mathcal{T}^T\mathcal{T}$:

$$\mathcal{T}^T\mathcal{T} = \frac{1}{Ddh^2} \mathcal{Q}_+ \mathcal{O}_+^T \mathcal{O}_+ \mathcal{Q}_+^T, \tag{73}$$

and computing $\mathcal{O}_+^T \mathcal{O}_+$:

$$\left[\mathcal{O}_+^T \mathcal{O}_+\right]_{kl} = \left[\mathcal{O}^T \mathcal{O}\right]_{kl} - 1 = \begin{cases} D - 1; & k = l \\ -\frac{D-1}{h-1}; & k \neq l \end{cases} \tag{74}$$

This $h \times h$ matrix has the eigenvalues:

1. $\lambda_0 =$ with multiplicity 1, where the eigenspace is spanned by $\mathbf{1} := (1, \ldots, 1)^T$; and -
2. $\lambda_1 = \frac{h(D-1)}{h-1}$ with multiplicity $h-1$ and eigenspace $\Lambda := (\text{span}\{\mathbf{1}\})^\perp$.

Next, we consider $\mathcal{Q}_+^T : \mathbb{R}^{d^2-1} \to \mathbb{R}^h$. This time it does not have an empty kernel. However, it turns out we need not show that $\text{Im}\, \mathcal{Q}_+^T = \Lambda$. It suffices to show $\text{Im}\, \mathcal{Q}_+^T \subset \Lambda$. Indeed, this follows simply as before:

$$\forall \boldsymbol{v} \in \mathbb{R}^{d^2-1}, \quad \mathbf{1} \cdot \mathcal{Q}_+^T \boldsymbol{v} = \mathcal{Q}_+ \mathbf{1} \cdot \boldsymbol{v} = \mathbf{0}, \tag{75}$$

where the last transition is just (69). Thus we obtain:

$$\mathcal{O}_+^T \mathcal{O}_+ \mathcal{Q}_+^T = \frac{h(D-1)}{h-1} \mathcal{Q}_+^T. \tag{76}$$

To conclude the proof, we must analyze $\mathcal{Q}_+ \mathcal{Q}_+^T$. Since for $h < d^2$ the projections $\{Q_k\}$ do not comprise a SIC-POVM, $\mathcal{Q}_+ \mathcal{Q}_+^T$ is no longer a frame operator of a tight frame, and thus not necessarily scalar. However, we may use the fact that $\mathcal{Q}_+ \mathcal{Q}_+^T$ and $\mathcal{Q}_+^T \mathcal{Q}_+$ have the same *nonzero* eigenvalues (i.e., squares of the singular values of $\mathcal{Q}_+$). Therefore, our next step would be computing $\mathcal{Q}_+^T \mathcal{Q}_+$:

$$\left[\mathcal{Q}_+^T \mathcal{Q}_+\right]_{kl} = \left[\mathcal{Q}^T \mathcal{Q}\right]_{kl} - 1 = \begin{cases} d-1; & k = l \\ -\frac{d-1}{h-1}; & k \neq l \end{cases} \tag{77}$$

As before, it is straightforward to note this $h \times h$ matrix has two eigenvalues: $\lambda_0 = 0$ with multiplicity 1, and $\lambda_1 = \frac{h(d-1)}{h-1}$ with multiplicity $h-1$. Thus, $\mathcal{Q}_+ \mathcal{Q}_+^T$ has the eigenvalues λ_1 with multiplicity $h-1$, and 0 with multiplicity $d^2 - h$.

To conclude, we observed the following:

1. $\mathcal{T}^T \mathcal{T} = \frac{1}{Ddh^2} \frac{h(D-1)}{h-1} \mathcal{Q}_+ \mathcal{Q}_+^T$,
2. $\mathcal{Q}_+ \mathcal{Q}_+^T$ has precisely $h-1$ nonzero eigenvalues, which all equal $\lambda_1 = \frac{h(d-1)}{h-1}$.

Thus, $\mathcal{T}^T \mathcal{T}$ also has $h-1$ nonzero eigenvalues, which all equal $\lambda' = \frac{D-1}{D(h-1)} \frac{d-1}{d(h-1)}$. Consequentially, the h largest singular values of $\mathcal{C}$ are $\sigma_0 = 1/\sqrt{Dd}$ with multiplicity 1, and $\sqrt{\lambda'}$ with multiplicity $h-1$; and the CMN is their product, that is:

$$\mathcal{M}_{h,p=\infty} = \frac{1}{\sqrt{Dd}} \left[\frac{D-1}{D(h-1)} \frac{d-1}{d(h-1)}\right]^{\frac{h-1}{2}} \tag{78}$$

which concludes the proof.

V. RELATION TO QUANTUM DISCORD

It is known [12] that for any given state ρ, the quantum discord $\mathcal{D}^A$ is zero if and only if there exists a local measurement on A that does not disturb the state. Here, a measurement corresponds to any orthonormal basis $\{\Pi_l\}$ of $\mathcal{H}_A$, where $\Pi_l = |l\rangle\langle l|$ and $\langle k|l\rangle = \delta_{kl}$. Measuring the state ρ in this basis transforms it by:

$$\rho \to \rho' = \sum_{l=1}^{d_A} (\Pi_l \otimes \mathbb{1})\, \rho\, (\Pi_l \otimes \mathbb{1}). \tag{79}$$

Let us find the transformation ondergone by the correlation matrix:

$$\begin{aligned} \mathcal{C}'_{ij} &= \text{tr}\left(\rho' A_i \otimes B_j\right) = \\ &= \sum_{l=1}^{d_A} \text{tr}\left((\Pi_l \otimes \mathbb{1})\, \rho\, (\Pi_l \otimes \mathbb{1})\, A_i \otimes B_j\right) = \\ &= \sum_{l=1}^{d_A} \text{tr}\left(\rho\, (\Pi_l A_i \Pi_l) \otimes B_j\right) = \sum_{l=1}^{d_A} \langle l|A_i|l\rangle\, \text{tr}\left(\rho \Pi_l \otimes B_j\right). \end{aligned} \tag{80}$$

Since $\{A_i\}$ comprise an orthonormal basis of the space of Hermitian matrices over $\mathcal{H}_A$, we may write:

$$\Pi_l = \sum_{k=1}^{d_A^2} \text{tr}\left(A_k \Pi_l\right) A_k = \sum_{k=1}^{d_A^2} \langle l|A_k|l\rangle\, A_k. \tag{81}$$

Plugging into (80), we obtain:

$$\mathcal{C}'_{ij} = \sum_{k,l=1}^{d_A^2} \langle l|A_i|l\rangle \langle l|A_k|l\rangle \underbrace{\text{tr}\left(\rho A_k \otimes B_j\right)}_{\mathcal{C}_{ij}}. \tag{82}$$

Hence, the post-measurement correlation matrix is given by $\mathcal{C}' = \mathcal{A}\mathcal{C}$, where $\mathcal{A}$ is a $d_A^2 \times d_A^2$ real matrix given by:

$$\mathcal{A}_{ik} := \sum_{l=1}^{d_A} \langle l|A_i|l\rangle \langle l|A_k|l\rangle. \tag{83}$$

Equivalently, $\mathcal{A}$ may be written as XX^T, where:

$$X_{il} = \langle l|A_i|l\rangle, \quad 1 \le i \le d_A^2,\, 1 \le l \le d_A. \tag{84}$$

Note that $\text{rank}\, X = d_A$, since its columns are the components of the orthonormal set $|l\rangle\langle l|$ in the orthonormal basis $\{A_i\}$ (w.r.t. the Hilbert-Schmidt inner product). In fact, this logic also shows that the columns of X form an orthonormal basis. Hence $X^T X = \mathbb{1}_{d_A}$, and we may observe that

$$\mathcal{A}^2 = X \underbrace{X^T X}_{\mathbb{1}} X^T = \mathcal{A}, \tag{85}$$

i.e. $\mathcal{A}$ is a rank-d_A projection matrix. However, not *every* $d_A^2 \times d_A^2$ projection matrix with rank d_A is obtained by this construction from some orthonormal basis for $\mathcal{H}_A$.

Note that the construction of $\mathcal{A}$ is exactly the same as in Theorem 1 of [13]. Since $\mathcal{M}_{h=1,p=2} = \text{tr}\left(CC^T\right)$ for any matrix C (this is the sum of squared singular values), it is clear that $\mathcal{D}_G^A = \mathcal{D}_{h=1,p=2}^A$.

Let us prove Theorem 5. First, by Theorem 6.7(7) in [14], for all $k \in \left\{1, \ldots, d_A^2\right\}$ we have

$$\sigma_k\left(\mathcal{AC}\right) \leq \left\|\mathcal{A}\right\|_1 \sigma_k\left(\mathcal{C}\right), \tag{86}$$

where $\left\|\mathcal{A}\right\|_1 = \max_j \sigma_j\left(\mathcal{A}\right) = 1$, as $\mathcal{A}$ is a projection. Therefore, $\sigma_k\left(\mathcal{AC}\right) \leq \sigma_k\left(\mathcal{C}\right)$ for all k, and we conclude that $\mathcal{M}_{h,p}\left(\rho\right) \geq \mathcal{M}_{h,p}\left(\rho'\right)$ for all h, p, using the fact that the CMNs are all monotonically non-decreasing w.r.t. the singular values σ_k.

Now, suppose ρ has zero discord. As we have already noted, there must be a measurement that does not disturb the state - i.e., there exists a matrix $\mathcal{A}$ such that $\mathcal{AC} = \mathcal{C}$. Then, for this choice of measurement, we have $\mathcal{M}_{h,p}\left(\rho\right) - \mathcal{M}_{h,p}\left(\rho'\right) = 0$. By the non-decreasing property for the CMN we have proven above, this is indeed the maximum, hence $\mathcal{D}_{h\leq 2,p}\left(\rho\right) = 0$.

To prove the converse, suppose there exists some measurement Π^A that changes the state but does not change the singular values. By Theorem 1 of [13], this implies the discord is zero. Thus, for any positive-discord state, at least one singular value of $\mathcal{AC}$ is strictly smaller than the corresponding one in $\mathcal{C}$. This would decrease any *nonzero* monomial of degree h in which it appears; and such a monomial always exists if there are at least h nonzero singular values. Since we are assuming the state has discord, the rank of $\mathcal{C}$ must be at least two (this can be observed, e.g. using the condition described in [15]). Thus, we have proven that $\mathcal{D}_{h\leq 2,p}\left(\rho\right) > 0$ for any positive-discord state ρ.

The family of states depicted in Figure 2 is given by [16]:

$$\rho\left(q, r\right) = \begin{bmatrix} 0 & 0 & 0 & 0 \\ 0 & q & -r\sqrt{q\left(1-q\right)} & 0 \\ 0 & -r\sqrt{q\left(1-q\right)} & 1-q & 0 \\ 0 & 0 & 0 & 0 \end{bmatrix}. \tag{87}$$

[1] G. Jaeger, M. A. Horne, and A. Shimony, Phys. Rev. A **48**, 1023 (1993).

[2] D. M. Greenberger and A. Yasin, Phys. Lett. A **128**, 391 (1988).

[3] G. Jaeger, A. Shimony, and L. Vaidman, Phys. Rev. A **51**, 54 (1995).

[4] B.-G. Englert, Phys. Rev. Lett. **77**, 2154 (1996).

[5] J. D. Franson, Phys. Rev. Lett. **62**, 2205 (1989).

[6] B. Y. Peled, A. Te'eni, D. Georgiev, E. Cohen, and A. Carmi, Appl. Sci. **10**, 792 (2020).

[7] R. A. Horn and C. R. Johnson, *Matrix analysis* (Cambridge university press, 2012).

[8] O. Gittsovich, O. Gühne, P. Hyllus, and J. Eisert, Phys. Rev. A **78**, 052319 (2008).

[9] J. I. de Vicente, Quantum Inf. Comput. **7**, 624–638 (2007).

[10] J.-L. Li and C.-F. Qiao, Sci. Rep. **8**, 1442 (2018).

[11] S. F. Waldron, *An introduction to finite tight frames* (Springer, 2018).

[12] A. Bera, T. Das, D. Sadhukhan, S. S. Roy, A. S. De, and U. Sen, Rep. Prog. Phys. **81**, 024001 (2017).

[13] S. Luo and S. Fu, Phys. Rev. A **82**, 034302 (2010).

[14] F. Hiai and D. Petz, *Introduction to matrix analysis and applications* (Springer Science & Business Media, 2014).

[15] B. Dakić, V. Vedral, and Č. Brukner, Phys. Rev. Lett. **105**, 190502 (2010).

[16] S. Virzì, E. Rebufello, A. Avella, F. Piacentini, M. Gramegna, I. R. Berchera, I. P. Degiovanni, and M. Genovese, Sci. Rep. **9**, 1 (2019).
